# Barcoded Hybrids of Extracellular Vesicles and Lipid Nanoparticles for Multiplexed Analysis of Tissue Distribution

**DOI:** 10.1002/advs.202407850

**Published:** 2025-01-16

**Authors:** Alena Ivanova, Renata Chalupska, Ana Filipa Louro, Mike Firth, Hernán González‐King Garibotti, Leif Hultin, Franziska Kohl, Elisa Lázaro‐Ibáñez, Julia Lindgren, Gentian Musa, Erik Oude Blenke, Andreia M. Silva, Louis Szeponik, Agnes Taylor, Ida Viken, Xiaoqin Wang, Karin Jennbacken, John Wiseman, Niek Dekker

**Affiliations:** ^1^ Discovery Biology, Discovery Sciences BioPharmaceuticals R&D, AstraZeneca Pepparedsleden 1 Mölndal 43150 Sweden; ^2^ Advanced Drug Delivery, Pharmaceutical Sciences BioPharmaceuticals R&D, AstraZeneca Pepparedsleden 1 Mölndal 43150 Sweden; ^3^ Data Sciences and Quantitative Biology, Discovery Sciences BioPharmaceuticals R&D, AstraZeneca Cambridge CB2 0AA UK; ^4^ Bioscience Cardiovascular, Research and Early Development, Cardiovascular, Renal and Metabolism (CVRM) BioPharmaceuticals R&D, AstraZeneca Pepparedsleden 1 Mölndal 43150 Sweden; ^5^ Clinical Pharmacology and Safety Science Imaging and Data Analytics BioPharmaceuticals R&D, AstraZeneca Pepparedsleden 1 Mölndal 43150 Sweden; ^6^ Centre for Genomics Research, Discovery Sciences BioPharmaceuticals R&D, AstraZeneca Pepparedsleden 1 Mölndal 43150 Sweden; ^7^ Department of Medical Biochemistry and Biophysics Karolinska Institutet Solnavägen 1 Solna Stockholm 171 77 Sweden

**Keywords:** barcoding, biodistribution, exosomes, extracellular vesicles, targeting

## Abstract

Targeted delivery of therapeutic agents is a persistent challenge in modern medicine. Recent efforts in this area have highlighted the utility of extracellular vesicles (EVs) as drug carriers, given that they naturally occur in bloodstream and tissues, and can be loaded with a wide range of therapeutic molecules. However, biodistribution and tissue tropism of EVs remain difficult to study systematically. Here, a multiplexed approach is developed for simultaneous tracking of EVs from various cell lines within a single in vivo experiment. EVs are used from 16 different cell lines, and through controlled fusion with lipid nanoparticles (LNPs) carrying single‐stranded DNA barcodes, uniquely barcoded hybrid EV particle (hEV) library is generated. These hEVs are combined for a multiplexed in vivo biodistribution profiling in mice, and discovered that HAP1‐derived hEVs demonstrated lung tropism, suggesting that these hEVs may be used for targeted drug delivery into lung tissue. To examine this possibility further, it is shown that HAP1 hEV loaded with Cre mRNA displayed functional delivery to the lungs. Overall, the barcoded hEV technology enables rapid profiling of biodistribution across EV cell sources, which is poised to improve throughput and extent of EV studies, while reducing the number of animals required for research.

## Graphical abstract

The study presents a method for the simultaneous analysis of the in vivo biodistribution of extracellular vesicles from different sources. The authors uniquely labeled the EVs isolated from 16 different cell lines via pH‐induced fusion with lipid nanoparticles carrying the ssDNA barcodes. Using this approach, they identified a cell source of EVs which appears to have an increased tropism for lungs.

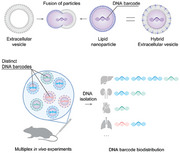

## 1 Introduction

Targeted drug delivery is a rapidly growing field that aims to improve the efficacy of drugs by directing them specifically to the site of action. Thus, targeted delivery has the potential to reduce the required therapeutic dose and minimize unwanted side effects.^[^ ^1^ ^]^ Despite its clear advantages, the development of targeted drug delivery systems remains a challenging task due to disease complexity and the need to address specific affected tissues or cells while avoiding healthy ones. Moreover, additional factors, such as presence of biological barriers in the body that hinder delivery (e.g., blood‐brain barrier),^[^ ^2^ ^]^ clearance activity of immune cells and excretory systems,^[^ ^3^ ^]^ the need for local controlled drug release,^[^ ^4^ ^]^ and the need to ensure biocompatibility and safety without causing immune responses or adverse reactions further exacerbate these challenges.^[^ ^5^ ^]^

Among targeted drug delivery systems, extracellular vesicles (EVs) are of growing interest, given that they are naturally occurring nanovesicles released by cells into the extracellular environment, and thus offer good biocompatibility.^[^ ^6^ ^]^ Additional advantages include their ability to transport both hydrophilic and hydrophobic molecules,^[^ ^7^ ^]^ and to target specific tissues.^[^ ^8^ ^]^ However, EV tissue specificity (i.e., tissue tropism) and how the cellular source of EVs governs biodistribution are currently incompletely understood, difficult to measure and study systematically.

Several studies have explored EV tropism for different tissues, as summarized by Kang et al.,^[^ ^9^ ^]^ but these studies have been hampered by the long and laborious procedures of EV isolation and labeling, and required individual testing in mice limiting the analysis to only few EV sources. In general, EVs have been observed to accumulate in organs with well‐developed phagocytic systems and high blood perfusions, such as the liver and spleen.^[^ ^10^ ^]^ However, the field is far from reporting full interaction maps between different types of EVs and their particular tropism. This is in part due to the difficulty of tracking EVs in vivo, which is primarily attributed to their complex composition, small size, and short half‐life in circulation.^[^ ^8^, ^11^ ^]^ While a few examples of optical, nuclear, and magnetic resonance imaging for in vivo tracking of EVs have been explored, monitoring EVs remains difficult.^[^ ^10^, ^11^, ^12^ ^]^ For example, while fluorescent and luminescent‐based imaging is the predominant method for biodistribution studies in small animals, it lacks the capacity to yield precise quantitative whole‐body biodistribution data due to the inaccuracy or low sensitivity of the method.^[^ ^11^ ^]^ Moreover, fluorescent‐based imaging has artifacts associated with labeling methods, particularly with lipophilic dyes, which can diffuse, leading to the tracking of the dye rather than the EVs.^[^ ^13^ ^]^ On the other hand, methods, such as PET or SPECT imaging, use radioisotopes that require highly specialized equipment and expertise with radioisotopes.^[^ ^11^, ^14^ ^]^ Importantly, none of the current methods allows for the study of several different types of EVs loaded with exogenous cargo at the same time and in the same animal. Studies like those are of special importance for rapid assessment of biodistribution as a function of the EV source, and for direct comparison of different EVs. Therefore, a user‐friendly method that would enable in vivo studies of EV biodistribution, preferably in a multiplex manner, would be highly enabling for the field.

To address this problem, we have developed a multiplexing strategy for monitoring and quantifying in vivo biodistribution of EVs. Our strategy combines the use of EVs from different cell lines with DNA barcodes that are delivered to the EVs via distinct lipid nanoparticles (LNPs). Thus, our new approach implements recent advances in barcoding, that is, the incorporation of unique identification markers into the delivery vehicles that allow multiple formulations to be delivered and tracked simultaneously within the same organism.^[^ ^15^ ^]^ Our protocol was optimized to use pH‐induced fusion between EVs and LNPs loaded with a DNA barcode cargo, producing hybrid EVs (hEVs). We employed hEVs from 16 different cell sources to explore the tropisms of hEVs simultaneously in a mouse model. We found that HAP1‐originated hEVs exhibited lung‐targeting potential, and confirmed that HAP1 hEVs loaded with Cre mRNA achieve functional delivery to the lungs in mice. Altogether, we showed that our method enables rapid acquisition of large biodistribution data sets, which can be used to map tissue tropism, and identify EVs that can serve as a starting point for the development of tissue‐specific drug delivery systems.

## 2 Results

### 2.1 LNPs can be Used for Loading EVs with DNA Barcodes

**Figure 1:**
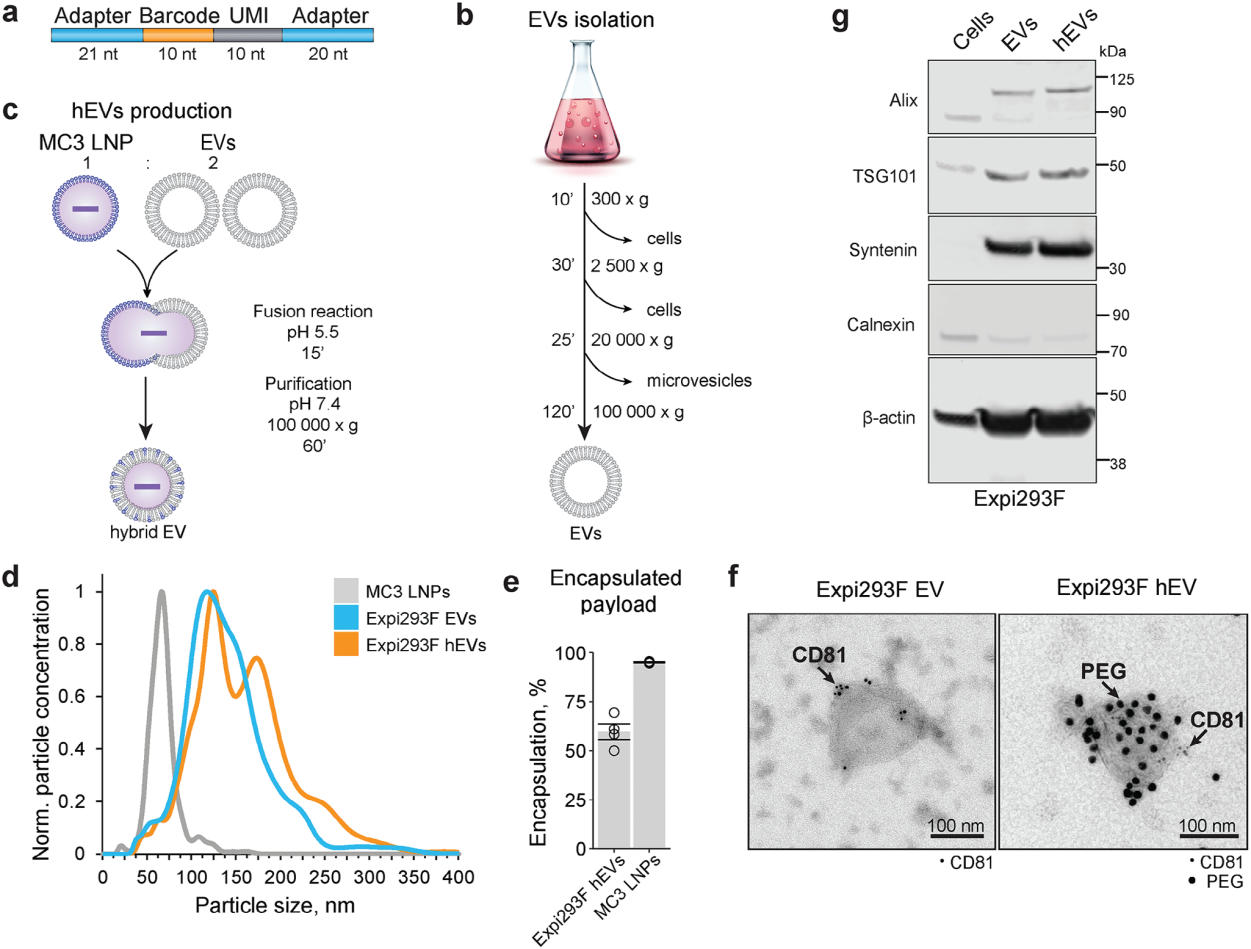
Production of hybrid extracellular vesicles (hEVs). a) DNA barcode structure. DNA barcode consists of a barcode region (orange), a unique molecular identifier (UMI) (grey), and 2 adaptor sequences for amplification (blue). b) EV isolation scheme. A differential centrifugation approach was used to isolate small EVs. First, cells were pelleted by two rounds of centrifugation at 300 x *g* and 2500 x *g*. Afterward, microvesicles were removed from the supernatant by centrifugation at 20 000 x *g*. Lastly, small EVs were pelleted at 100 000 x *g*. c) Schematic representation of hybrid EV production. EVs and respective LNPs were mixed at 2:1 ratio and kept in the fusion buffer at pH 5.5 for 15 min. Afterward, pH was adjusted to neutral and hEVs were pelleted down by ultracentrifugation at 100 000 x *g* for 60 min. d) Nanoparticle tracking analysis of LNPs (grey line), Expi293F EVs (dark blue line), and hEVs (blue line). A graph with a representative size distribution (y‐axis) versus particle concentration (x‐axis) is shown. e) Percentage of encapsulated payload in the produced Expi293F hEVs and MC3 LNPs. Plots show mean ± s.e.m. for 4 measurements for each condition. f) Transmission electron microscopy images of CD81 and PEG immunogold labeling of Expi293F‐derived EVs and hEVs. EVs were incubated with primary followed by secondary antibodies conjugated with 6 nm gold particles (for CD81). hEVs were additionally incubated with primary antibodies against PEG, and a secondary antibody conjugated with 15 nm gold particles (for PEG). Scale bar is = 100 nm. g) Representative western blot analysis of Alix, TSG101, Syntenin, Calnexin, and β‐actin in protein extracts from Expi293F EVs, Expi293F hEVs, and their parental cells. An equal amount of protein was loaded per lane.

As a proof‐of‐concept experiment, we used EVs from Expi293F suspension cells as a model and tested whether they can be loaded with DNA barcodes via fusion with LNP. In our experiment, we used DNA barcodes that were 61 nucleotides long, and consisted of a variable barcode sequence with a Unique Molecular Identifier (UMI), which enabled identification and quantification, and adapter regions for amplification and sequencing (**Figure** **1**a). We collected EVs using a differential centrifugation^[^ ^11^ ^]^ (Figure 1b). To load EVs with DNA barcodes we developed a pH‐dependent approach of controlled fusion of EVs with DLin‐MC3‐DMA (MC3) LNPs (Figure 1c), whereby the acidic environment triggers fusogenic instability of the LNP particles, resulting in the fusion with EVs. We maintained a 2:1 ratio between EV and LNP particles to favor the fusion of LNPs with EVs over the non‐productive LNP‐LNP fusions. After the acidic incubation, the pH was adjusted to neutral, and the resulting particles were pelleted by ultracentrifugation to isolate dense particles resulting from the fusion events of EVs and LNPs (thereinafter referred to as hybrid EVs or hEVs) (Figure 1c).

We evaluated the size distribution of EVs, hEVs, and LNPs by nanoparticle tracking analysis (NTA) (Figure 1d, Supplementary Figure S1a, Supporting Information). EVs and LNPs had an average size of 117 nm and 67 nm, respectively, while HEVs displayed an additional distinct population at ∼174 nm. The appearance of the second peak of larger‐sized particles suggested that there was an efficient fusion event between LNP and EV particles during the production of hEVs (Figure 1d). Next, we examined whether the LNP‐containing DNA cargo was successfully introduced in the hEVs as a result of the fusion, and observed 60% encapsulation efficiency in hEVs (compared to > 98% encapsulation efficiency in LNPs; Figure 1e). Thus, the fusion between EVs and LNPs resulted in single‐stranded DNA (ssDNA) loaded hEVs.

To further characterize hEVs we performed negative staining transmission electron microscopy (TEM) coupled with immunogold labeling of hEVs. We demonstrated the presence of EV markers CD81 and CD63 on the same particles where the LNP component polyethylene glycol (PEG) was detected (Figure 1f; Figure S1b,c, Supporting Information). Furthermore, EVs from Expi293F cells, hEVs, and the corresponding cell lysates were analyzed by Western blotting for the presence of markers of endosomal origin that are expected to be enriched in EVs. As expected, we detected enrichment of the canonical EV proteins Alix, TSG101, and Syntenin, together with β‐actin in both EVs and hEVs (Figure 1g). Importantly, we observed minimal presence of the endoplasmic reticulum (ER) protein marker calnexin in EV and hEV samples, indicating the absence of intracellular vesicles associated with the ER in the preparations.

Next, we examined whether LNP particles remained in the hEV preparations and could potentially contaminate the samples. We first tested if LNPs could be pelleted by ultracentrifugation under the same conditions used for hEV preparation (Figure S1d, Supporting Information). We pelleted 4.05×10^10^ MC3 LNPs carrying Cy5‐labeled mRNA. Switching to another cargo was required for Cy5 labeling and particle characterization and provided the initial data toward the goal of delivering therapeutically relevant mRNA cargo. The amount of recovered particles was at the detection limit of NanoAnalyzer flow cytometer, and no Cy5+ particles were detected. In contrast, when we mixed Expi293F EVs with MC3 LNPs at a 2:1 ratio, as for hEV preparations, and pelleted the mixtures, we detected 2% of Cy5+ particles in the resulting pellets, corresponding to MC3 LNPs (Figure S1e, Supporting Information). This indicates that less than 1% of the input MC3 LNPs were recovered. We then assessed the stability of MC3 LNPs in MES buffer. We incubated MC3 LNPs carrying Cy5‐labeled mRNA in MES buffer for 30 min, as done for hEV preparations, and measured particle concentration in the solution post‐incubation (Figure S1f, Supporting Information). A significant reduction in particle number was observed, suggesting that MC3 LNPs are not stable in MES buffer, with less than 3% of particles surviving the incubation. Finally, we examined the composition of the hEV samples at the single‐particle level (Figure S1g, Supporting Information). Expi293F EVs were labeled with PE‐TopFluoro AF488 dye and hEVs were prepared by fusing these EVs with MC3 LNPs carrying Cy5‐labeled mRNA, following the described hEV preparation protocol. Among the particles, 57% of EVs incorporated the PE‐TopFluoro AF488 dye, and 93% of LNPs were Cy5+. The fact that not all particles in the EV sample were labeled with the dye may indicate the presence of contaminations in the sample or reflect the limited efficacy of the dye in labeling all EVs. In the hEV sample, 33% of the particles were AF488‐/Cy5‐, corresponding to unlabeled EVs and LNPs. The AF488+/Cy5+ particles, representing hEVs resulting from the fusion of labeled EVs and LNPs, accounted for 23%. The AF488+ particles were labeled EVs (44%), and the Cy5+ particles were residual LNPs (0.2%). This analysis suggests that 0.2% of the particles in the hEV sample were LNPs. However, it is important to consider that unbound membrane dye and free‐labeled mRNA remained in the samples despite several washing steps. This residual presence could potentially lead to additional labeling of the particles, resulting in an overestimation of the AF488+, Cy5+ and double‐positive AF488+/Cy5+ particles. Nevertheless, together with previous results showing that less than 1% of LNPs are recovered after pelleting with EVs (Figure S1e, Supporting Information) and less than 3% of LNPs survive incubation in MES buffer (Figure S1f, Supporting Information), we conclude that the majority of particles in the hEV samples are unfused EVs and particles resulting from the fusion of EVs and LNPs, with a very minor contribution from unfused LNPs.

Additionally, to ensure the feasibility of scaling up hEV production, we compared the mRNA encapsulation efficiency in hEVs produced using EVs purified by two different techniques: tangential flow filtration (TFF) and ultracentrifugation (UC) (Figure S1h, Supporting Information). While using Cy5‐labeled mRNA, we observed a lower encapsulation efficiency (20% compared to 60%) compared to DNA oligos (Figure 1e), potentially due to the larger size of the mRNA cargo. Our results showed that TFF, a technique compatible with large‐scale production, demonstrated better encapsulation efficiency in the resulting hEVs compared to the use of EVs purified by ultracentrifugation.

In conclusion, these results demonstrate that we could successfully generate hEVs and load them with DNA cargo through fusion with LNPs.

### 2.2 EVs from Different Sources can be Uniquely Tagged using LNPs Loaded with Specific DNA Barcodes

**Figure 2:**
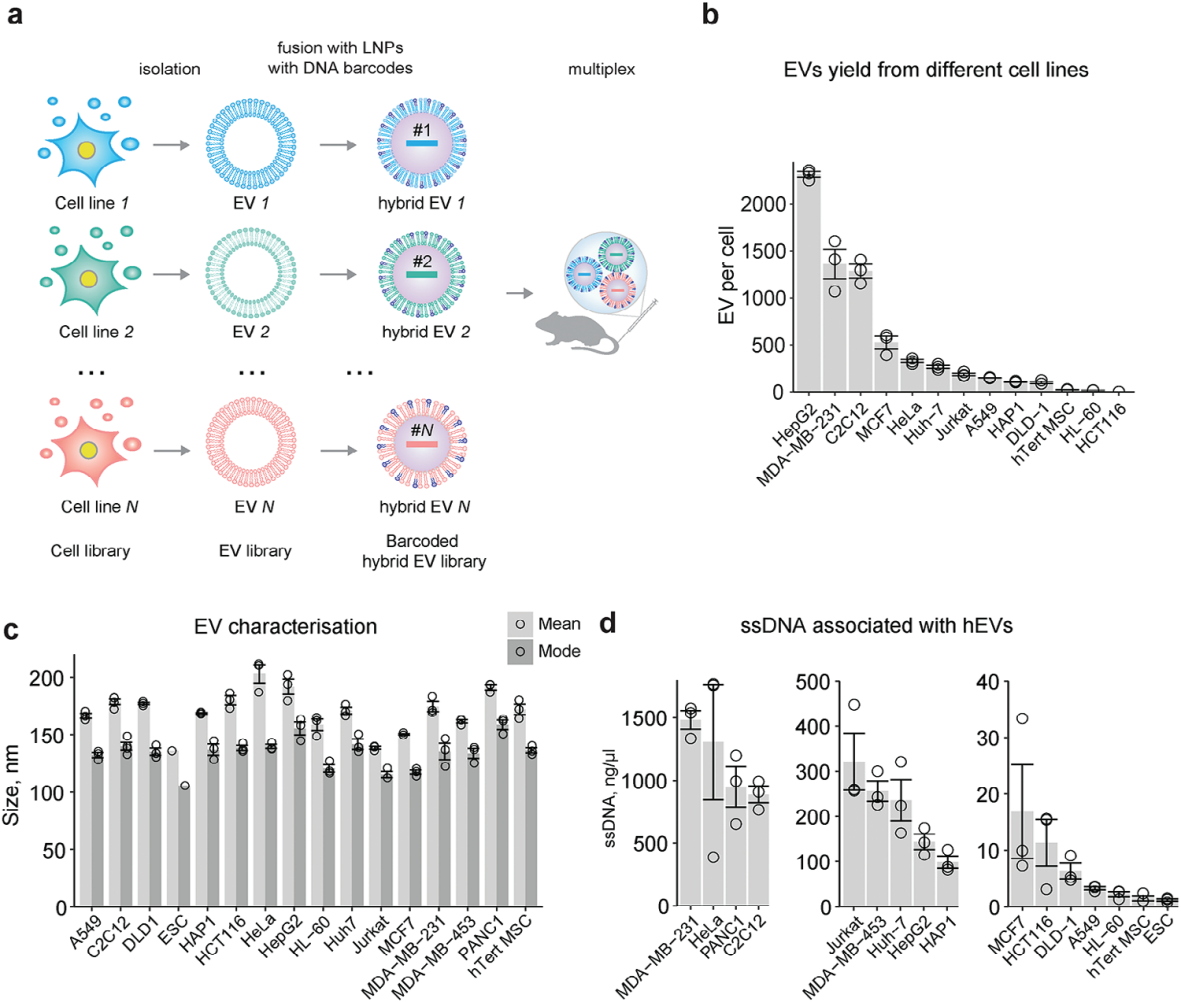
Preparing hEVs from different cell lines for multiplexing in vivo biodistribution experiments. a) Graphical summary describing EVs multiplexing approach. EVs from different cell lines were collected and fused separately with LNPs carrying a specific DNA barcode. After the fusion hEV particles from different cell lines loaded with the different DNA barcodes were purified and mixed together for the intravenous administration in mice. b) The EV yield from different cell lines is indicated. The cell culture media was changed to EV‐depleted media 24 h prior to EV isolation and EVs were collected as described in Figure 1A. Average number of EVs per cell measured after EV isolation ± s.e.m. is shown. c) Mean and mode size of the EVs collected from different cell lines. The size was calculated based on NTA particle analysis. Shown is an average of 3 measurements ± s.e.m. d) Characterisation of hEVs. The concentration of ssDNA associated with the particles was measured. Mean ± s.e.m. of 3 measurements is shown.

Next, we aimed to barcode the large set of EVs originating from different cell lines to study their biodistribution in vivo via a multiplex approach outlined in **Figure** **2**a. We chose 16 cell lines originating from 9 different tissues: liver (Huh‐7, HepG2), lung (A549), pancreas (PANC1), colon (DLD‐1, HCT116), muscle (C2C12), blood (HL‐60, Jurkat, HAP1), breast (MDA‐MB‐231, MCF7, MDA‐MB‐453), cervix (HeLa), and stem cells (telomerized human mesenchymal stem cells (hTert MSC), Human ESC Cell Line 121 (SA121) referred to as ESC). These cells were chosen because they represent a diverse set of tissues, grow relatively fast, and require simple media composition.

To characterize the EV production capacity of the chosen cell lines, we calculated EV secretion yields at the time of EV collection (apart from MDA‐MB‐453, PANC1, and ESC) (Figure 2b; Figure S2a,b, Supporting Information). Unexpectedly, we could not observe any correlation between cell number and EV yield. For example, although HCT116 cells reached confluency with the highest cell number, they did not produce the highest amount of EVs (Figure 2b; Figure S2b, Supporting Information); whereas HepG2 cells, which displayed lower cell numbers, produced the most EVs per cell, as well as per the supernatant volume (Figure 2b; Figure S2a, Supporting Information). All produced EVs were characterized by NTA, which showed that the EVs were of relatively uniform size (mean: 136 nm – 203; mode: 101 nm – 155 nm; Figure 2c), all falling within the size range for the EVs.

Isolated EVs were used for the production of hEVs following our standard procedure. TEM with immunogold labeling confirmed the formation of hEVs, demonstrating the presence of both CD81 EV marker and LNP PEG component on the same particles, in the produced hEVs (Figure S2c,d, Supporting Information). We observed a range of DNA barcode loading efficiencies across hEVs from different cell lines irrespective of the input number of particles for the reactions (Figure 2d; Table S1, Supporting Information). For example, although the starting quantity of HeLa EVs was high, we could not detect ssDNA inside the resulting hEV. In contrast, we observed the opposite for DLD‐1 EVs, which displayed high encapsulation efficiency despite low EV numbers in the fusion reaction (75%; Figure S2e, Supporting Information). For A549, HL‐60, and ESC encapsulation efficiency was 100% (Figure S2e, Supporting Information). The differences in barcode loading suggest that the surface composition of EVs and possibly the protein corona may affect the propensity for fusions.

Next, we evaluated the stability of hEV particles and their potential to fuse with each other during storage. We tested Expi293F and HAP1 hEVs. We prepared two types of particle mixes. We used a 1:1 mixture of Expi293F hEVs, with 78% labeled using PE‐TopFluoro AF488 dye, and HAP1 hEVs, with 35% labeled with Cy5 (Figure S2f, Supporting Information). After mixing the theoretical percentage of AF488+ particles would be 39%, which is very close to the observed level of 37.6% after 16 h of co‐incubation at +4 °C (Figure S2f,g, Supporting Information). This suggests that the particles remained stable during the incubation time. We did observe 2.4% of double‐labeled particles, indicating less than 10% fusion and minimal scrambling of the label. For the mixture of the same type of particles, HAP1 hEVs were labeled with either AF488 (95% labeled) or Cy5 (35% labeled) (Figure S2f, Supporting Information). After 16 h at +4 °C, we noted a slightly higher fusion level, with 7.2% double‐labeled particles (Figure S2f,g, Supporting Information), suggesting some intrinsic interactions among identical particles, which could be due to intrinsic homing. It is important to note that unbound membrane dye and free labeled mRNA present in the hEV mixture can lead to an overestimation of double‐positive particles, as dye and free mRNA can interact with unstained particles. Overall, these results suggest that fusion events between different hEV particles may depend on the specific hEV type. We concluded that there was no risk of significant exchange of the DNA barcodes in the multiplex experiments due to particle fusion in the mix prior to injection to the animal.

Taken together, these results show that we successfully generated hybrid particles that combine properties of EVs, such as surface molecules that are responsible for the tissue tropism, and LNPs, such as DNA payload and additional (ionizable) lipids that facilitate endosomal escape.

### 2.3 DNA Barcoded hEVs Facilitate High‐Throughput In Vivo Biodistribution Analysis

**Figure 3:**
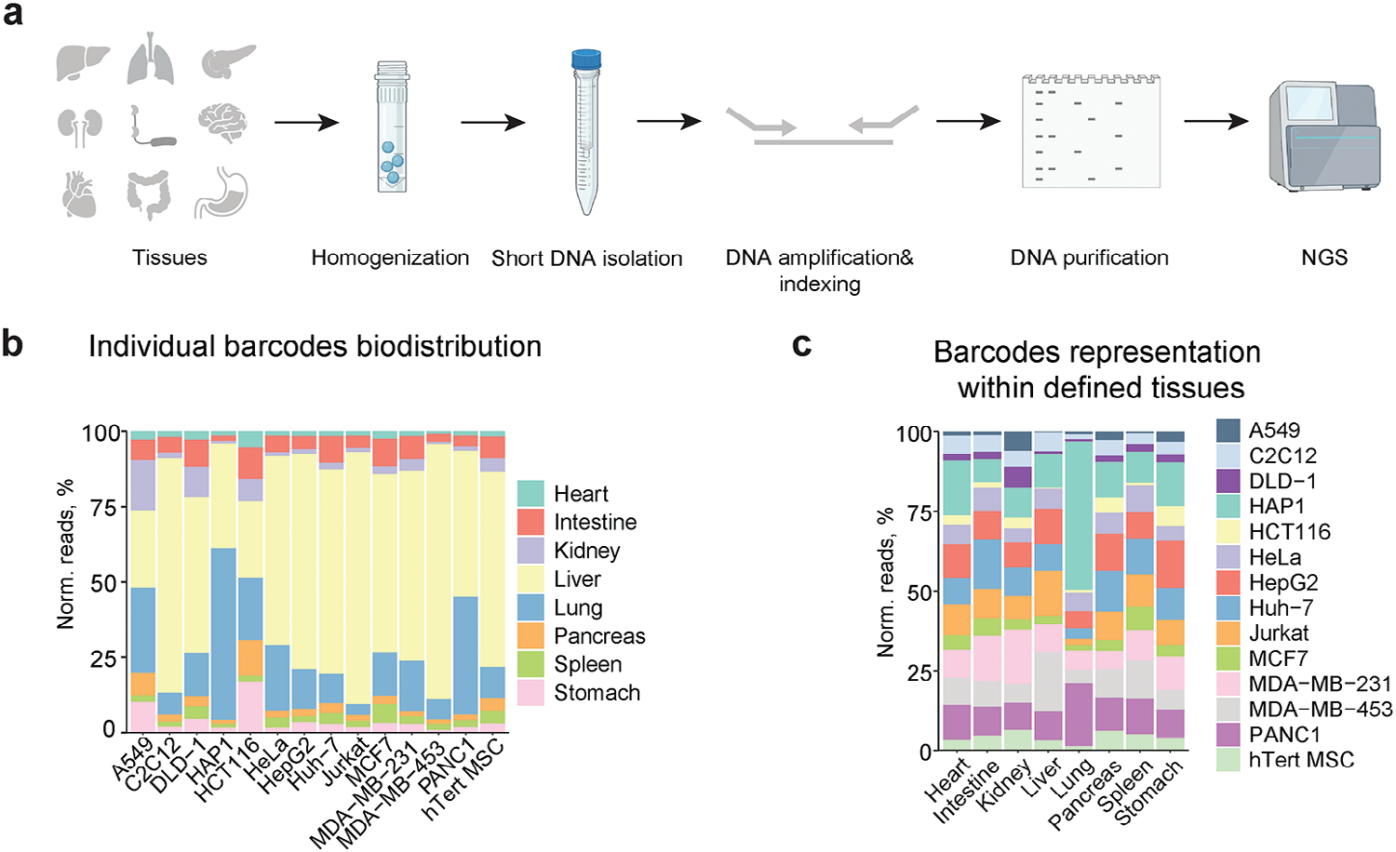
In vivo biodistribution of hEVs as assessed by barcode detection in distinct organs. a) Schematic representation of barcode isolation from different organs for further amplification and indexing for NGS. After homogenization, short DNA fragments were isolated from the tissue lysates and amplified with primers containing indexes for NGS. PCR products were purified and separated from primer dimers following agarose gel electrophoresis. Purified amplicons were sequenced on the Illumina MiSeq instrument. b) Individual barcode biodistribution across examined tissues. The total number of normalized unique counts for each barcode was set as 100%. c) Barcode representation within defined tissues. All normalized unique counts identified within the tissue were set as 100%.

To examine whether our hEVs can facilitate multiplexed EV biodistribution studies, we pooled all hEVs (Table S2, Supporting Information) and injected them into mice via the tail vein. One hour after intravenous injection, brain, heart, intestine, kidney, liver, lung, pancreas, spleen, and stomach were collected and homogenized. We used tailored homogenization protocols for the various tissues depending on the tissue structure to ensure similar levels of homogenization (**Figure** **3**a). We recovered DNA barcode oligonucleotides and prepared the samples for the NGS (see Methods; Figure S3a, Supporting Information). We were able to detect barcodes corresponding to particles injected at 2 × 10^−5^ mg kg^−1^ or higher (Figure S3b, Supporting Information). We calculated the percentage of retrieved barcodes from the organs, and observed a 92% recovery rate for HAP1 hEVs, followed by PANC1 hEVs with 51% (Figure S3b, Supporting Information).

Next, we investigated the biodistribution of individual barcodes (Figure 3b). For this purpose, we normalized the number of reads corresponding to a barcode found in the tissue to the size of the processed tissue. We set the sum of the normalized reads for the same barcode to 100%, which allowed us to map biodistribution of a given barcode across different tissues. As expected, the liver was a major target organ for most of the tested particles (Figure 3b; Figure S3c,d, Supporting Information). Interestingly, we observed that HAP1 and A549 hEV barcodes exhibited a preferred distribution to the lungs, with 57% of normalized reads for HAP1 EV found in the lungs, as compared to 35% in the liver (Figure 3b; Figure S3c, Supporting Information). This difference was less pronounced for A549, with 28% and 26% of normalized reads found in the lungs and liver, respectively (Figure 3b; Figure S3c, Supporting Information). In general, brain and heart tissues were the most difficult‐to‐reach for all the tested particles, with no barcodes detected in the brain and very small amounts detected in the heart tissue.

Next, we compared the accumulation of different barcodes within the same organ. Here, we first normalized the number of reads corresponding to barcodes found in the tissue to the number of the barcode reads in the input library. Then the sum of the normalized reads of all barcodes in a given organ was set as 100%. This allowed us to compare barcode distribution within individual organs taking into account the differences in injection dose for different hEV preparations. The major portion of the barcodes detected in lungs corresponds to HAP1 hEVs (46%), suggesting that HAP1 hEVs have the strongest lung tropism among all the examined particles (Figure S3d, Supporting Information).

Taken together, the designed approach allowed us to acquire biodistribution data for hEV particles and, at the same time, compare their tissue tropism in the same animal. Importantly, this allowed us to identify HAP1 hEVs as exhibiting prominent lung‐targeting properties. Therefore, we chose to test HAP1 hEVs as a vehicle for functional mRNA delivery to the lungs.

### 2.4 HAP1 hEVs Achieve Functional Delivery of mRNAs to the Lungs

**Figure 4:**
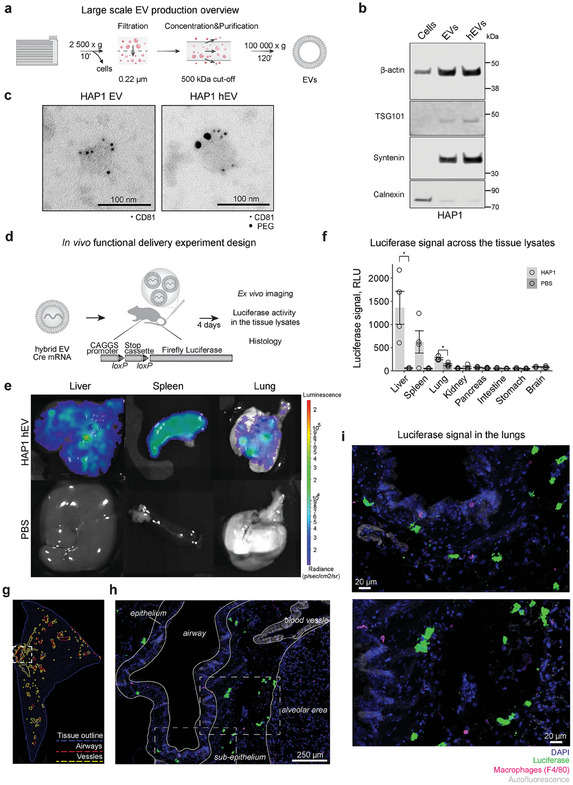
Cre mRNA functional delivery in vivo with HAP1 hEVs. a) Large‐scale EV production overview. b) Representative western blot analysis of TSG101, Syntenin, β‐actin, Calnexin in protein extracts from HAP1 EVs, HAP1 hEVs, and their parental cells. Equal amount of protein was loaded per lane. c) Transmission electron microscopy images of CD81 and PEG immunogold labeling of HAP1 EVs and HAP1 hEVs. EVs were incubated with primary antibodies against CD81, followed by secondary antibodies conjugated with 6 nm gold particles. hEVs were additionally incubated with primary antibodies against PEG, followed by secondary antibodies conjugated with 15 nm gold particles. Scale bar is = 100 nm. d) In vivo functional delivery experimental design. Cre reporter animal model harbors a stop signal between *loxP* sites preventing luciferase expression. Upon release of Cre mRNA into the cells, Cre recombinase is produced, resulting in the removal of the stop signal between *loxP* sites and enabling luciferase expression. Ex vivo imaging was performed four days after hEVs injections. e) Representative ex vivo images of the liver, spleen, and lung of HAP1 hEV‐ and PBS‐treated animals. f) Quantitative organ biodistribution profile from tissue lysates of mice treated with HAP1 hEVs or PBS. Organs were homogenized in lysis buffer and cleared of tissue debris. The same amount of tissue lysates was used for luciferase signal detection. Relative luciferase signal (mean ± s.e.m.) is shown (*n* = 4). *p*‐value was calculated using the one‐way ANOVA test. Asterisks indicate that *p* < 0.05. g–i) Representative immunohistology images of lung tissue from the animals treated with HAP1 hEVs. g) Lung tissue section. Dotted lines indicate tissue outline (blue), airways (red), and blood vessels (yellow). The magnification of the region in the white rectangle is shown in h). h) Lung tissue stained with the DAPI nuclear dye (blue), anti‐luciferase antibody (green), and anti‐F4/80 antibody to detect macrophages (magenta). Tissue autofluorescence (grey) was used to identify blood vessels. The lung airway is surrounded by epithelial cells, between epithelial cells and the alveolar region lies the sub‐epithelium. Magnifications of the regions in the white rectangles are shown in i). Scale bar is = 250 µm. i) Localization of luciferase positive cells and macrophages in sub‐epithelium and epithelium lung regions. Scale bar is = 20 µm.

Considering the low EV yield obtained from HAP1 cells (Figure 2b), we scaled‐up HAP1 EVs production using tangential flow filtration (TFF) (**Figure** **4**a). Freshly isolated EVs were fused with LNPs loaded with Cre mRNA and resulting HAP1 hEV hybrid particles were characterized by Western blotting (Figure 4b), NTA (Figure S4a,b, Supporting Information), and TEM (Figure 4c; Figure S4c, Supporting Information). We observed the expected increase in the mode size of particles from 127 to 139 nm after fusion with LNPs (Figure S4a,b, Supporting Information) indicative of successful fusion. EV and hEV fractions were enriched for β‐actin together with TSG101, and Syntenin compared to parental cell lysate, indicating their endosomal origin. Calnexin was hardly detected in EV and hEV samples, demonstrating their purity (Figure 4b). TEM images revealed the presence of hEV particles that contained both EV marker CD81 and LNP component PEG. Lastly, our HAP1 hEVs achieved ∼20% mRNA encapsulation efficiency, while the average LNP encapsulation efficiency was ∼92% (Figure S4d, Supporting Information). Altogether these results demonstrated that we successfully fused HAP1 EVs with mRNA‐containing LNPs in a manner that retains mRNA cargo encapsulation.

To evaluate HAP1 hEVs functional delivery capacity in vivo, we loaded them with Cre mRNA and administrated them to Cre reporter animals at 135 µg total RNA kg^−1^ HAP1 hEV (Figure 4d). Then, we administered D‐luciferin 4 days after loaded HAP1 hEV delivery, and performed ex vivo imaging of liver, spleen, lung, kidney, pancreas, intestine, stomach, and brain. We observed bioluminescence due to luciferase activity in liver, spleen, and lungs (Figure 4e). We performed a quantitative analysis of the luciferase activity in tissue lysates and observed the highest activity in liver, followed by spleen and lungs (Figure 4f). Despite high tropism to the lung tissue observed in the previous experiments, lung functional delivery levels were lower compared to liver.

To dissect factors that affect functional delivery of mRNAs to the lungs, we performed immunohistological analyses of the lungs to investigate which cell type(s) were responsible for the uptake of hEVs. We also examined the potential contribution of hEV aggregates trapped in the fine vascular system to the higher concentration of hEVs observed in the lungs. Lung sections were stained with nuclear dye and tissue autofluorescence was used to detect blood vessels. We could clearly observe the various lung structures such as airways, epithelial cells surrounding airways, blood vessels, the alveolar area of the lungs, and the sub‐epithelium region (Figure 4g,h; Figure S4e,f, Supporting Information). We used an antibody against firefly luciferase to identify cells that had taken up hEVs capable of functional Cre delivery. We also stained macrophages using F4/80 antibody since EVs are often cleared by these cells.^[^ ^6a^ ^]^ We did not observe luciferase‐positive cells near the small blood vessels; in contrast, luciferase signal was close to the big blood vessels (Figure 4h,i). This suggests that HAP1 hEVs leave the bloodstream and penetrate lung tissue by a distance of several cell diameters. Most of the cells that expressed luciferase were found in the sub‐epithelium region, which is rich in immune cells and extracellular matrix. We did not observe luciferase‐positive F4/80 macrophages, suggesting that other cell types are responsible for hEVs uptake. We confirmed that HAP1 hEVs display lung tropism, albeit the functional delivery levels to the lung cells are 5.2 times lower compared to liver cells (Figure 4f). Therefore, further optimization of HAP1 hEV properties will be required to improve targeted delivery to lungs. Nevertheless, taken together, these results demonstrate the utility of combining EVs with LNP barcoding for rapid investigation of hEV biodistribution and identification of hEVs with the highest potential for achieving targeted drug delivery.

## 3 Discussion

The use of EVs as delivery vehicles for therapeutic drugs has been of growing interest, and EV‐based formulations have reached clinical testing.^[^ ^16^ ^]^ However, the analysis of exogenously loaded EV biodistribution remains laborious and typically involves one group of animals for every single experimental condition, significantly increasing the number of animals required, which in turn has a negative environmental impact and is associated with increased resource consumption and waste. Here, we addressed these problems by developing a multiplexing technique that combines the use of EVs with the use of DNA barcodes to enable simultaneous analysis of multiple samples in a single animal, and provide high sensitivity of particle detection in the tissues.

In multiplexed experiments, a crucial development was our capability to load EVs with DNA barcodes. Currently, there are two primary strategies for loading cargo into EVs: endogenous and exogenous loading. Endogenous loading involves genetic modification of cells to facilitate the transfer of cargo to secreted EVs, but challenges persist in efficiently packaging cargo into EVs.^[^ ^17^ ^]^ Notably, popular exogenous loading methods, such as electroporation and sonication, have demonstrated limitations in loading efficiency and potential impacts on EV integrity and function.^[^ ^18^ ^]^ To address these challenges, we performed a fusion of EVs and LNPs using pH changes to direct the fusion process. This allowed us to load EVs and create hybrid particles containing short single‐stranded DNA molecules. This approach was successfully applied across a wide range of cell lines, revealing variations in fusion efficiency based on the origin of the EVs. The surface composition of EVs including membrane lipids and surface proteins as well as the protein corona that is formed around the particles in the culture media may be responsible for these differences.^[^ ^19^ ^]^ Our findings underscore the need for future refinement of this method to ensure efficient loading of EVs from diverse cell origins. Adjustments of parameters such as the pH of the fusion buffer, fusion incubation time, and lipid composition of LNPs based on the specific EV origin can potentially further improve cargo loading efficiency. Nevertheless, we demonstrated that the fusion of EV and LNP enabled the loading of a sufficient number of barcodes to the hEVs allowing us to investigate the biodistribution of these particles in vivo.

In this study, we chose to employ the MC3 ionizable lipid in our LNP formulation due to its extensive documentation and well‐established status, making it a reliable benchmark even in the face of newer lipids emerging for LNP composition.^[^ ^20^ ^]^ However, it is important to note that MC3 LNPs tend to accumulate primarily in the liver,^[^ ^21^ ^]^ posing a potential challenge for achieving effective extrahepatic delivery. Implementation of LNPs with lipids with “neutral” biodistribution may lead to a less biased assessment of delivery outcomes. Additionally, pegylation of EVs is known to affect their biodistribution,^[^ ^22^ ^]^ which provides opportunities for engineering extrahepatic hEVs by incorporating LNPs with relevant PEG‐lipids. These aspects might open up new avenues for future research to optimize and tailor hEV biodistribution for specific therapeutic applications.

The use of NGS techniques was also instrumental in achieving high sensitivity in the detection of hEVs in tissue samples. We detected DNA barcodes corresponding to particles injected at 2 × 10^−5^ mg kg^−1^ or higher dose, which means that for ESC and HL‐60 hEVs (10^−5^ mg kg^−1^) were not retrieved. Going forward, one of the possible solutions to increase the sensitivity of the assay is to perform deeper sequencing, which can be easily implemented. Nevertheless, we cannot exclude the possibility that some of the barcodes are degraded or ended up in the tissues that were not examined, or that particles were phagocytized by immune cells in the blood and therefore were not able to reach target organs. Future studies are needed to examine these possibilities and further optimize the system.

Importantly, our results are in general agreement with the existing literature. For example, we demonstrate that the liver is one of the main target organs for the majority of tested hEVs, which is in line with the previous reports demonstrating that EVs end up in the organs with well‐developed phagocytic systems and high blood perfusions, such as the liver.^[^ ^10a^ ^]^ Additionally, C2C12 and MDA‐MB‐231 hEVs recapitulated previous biodistribution reports.^[^ ^10^, ^23^ ^]^ Interestingly, we discovered that hEVs originated from HAP1 cells displayed lung tropism. To eliminate the possibility that lung tropism was driven by aggregation, we performed immunohistochemistry analysis of the lung tissues. We did not observe HAP1 hEV uptake by the cells near the small blood vessels, in contrast, we observed luciferase signals close to the big blood vessels. This suggests that HAP1 hEVs leave the bloodstream and penetrate the tissue by more specific mechanisms rather than mechanical accumulation in the fine vascular structures. We observed that HAP1 hEVs accumulated in the sub‐epithelium lung tissue and did not release their content in cells within the alveolar space. This highlights the importance of evaluating the biodistribution at the cellular level. Further investigation of the HAP1 EV surface composition might shed light on the nature of this tropism as it was previously reported that integrin molecules help to bring different modalities to the lungs.^[^ ^24^ ^]^

Regardless of a strong tropism of HAP1 hEVs to the lungs, we observed limited levels of Cre mRNA functional delivery to the lung cells. This might indicate that despite the high lung tissue tropism, HAP1 hEV particles experience difficulties in releasing their content in the lung cells. Nevertheless, although limited, the lung signal we obtained is substantially higher than the unspecific lung‐associated signals detected in the previous studies.^[^ ^11^ ^]^ We hypothesize that after internalization, the majority of hEVs were trapped in endosomes and degraded or recycled in lysosomes, which limits the delivery of cargo into the cytoplasm.^[^ ^25^ ^]^ In the case of EVs that were not fused with LNPs, combination treatment with EVs and an endosomal escape enhancer was required to achieve EV‐mediated functional delivery, reinforcing the idea that endosomal escape is a further bottleneck for the development of EV‐based delivery systems.^[^ ^6a^ ^]^ In this study, ionizable lipids in hEVs possibly partly helped the particles escape endosomes. Nevertheless, the combination of EV and LNP‐related molecules in hEVs needs to be further optimized to create particles with targeting properties of EVs and facilitate robust endosomal escape as typical of LNPs.

Additionally, the differences observed between biodistribution and functional delivery studies may be linked to the hypothesis that in vivo delivery of one type of hEV is influenced by the presence of other hEVs within the pool. In other words, if a particular hEV demonstrates a high affinity for the liver, it may predominantly interact with hepatic cells, potentially allowing other hEVs with distinct tropisms to circulate more freely throughout the body in a manner aligned with their biological behaviors. This dynamic interaction among hEVs could potentially impact their biodistribution patterns and functional delivery. Notably, a similar principle was observed in prior studies where the pre‐injection of liposomes in mice served to “occupy” the liver, enabling other substances to distribute more extensively.^[^ ^26^ ^]^ This dynamic interplay among hEVs and their differing affinities for specific tissues might be important to consider in designing libraries for multiplex biodistribution studies.

## 4 Conclusion

Overall, DNA barcoding nanocarriers represent a powerful and highly sensitive tool for advancing our understanding of targeted delivery and for developing more effective and efficient drug delivery systems. The implementation of multiplexing technology also promotes ethical and humane research practices.

## 5 Experimental Section

### Cell Culture

Expi293F cells were cultured in Expi293 expression media (A1435101, ThermoFisher) according to manufacturer recommendations at 37 °C in 8% CO_2_.

A549, C2C12, HeLa, HepG2, Huh‐7, MCF7, MDA‐MB‐231, MDA‐MB‐453, PANC1, and hTert MSC were cultured in high glucose DMEM (11965092, Gibco), 2 mM glutamine, and 0.1 mM MEM Non‐Essential Amino Acids; Jurkat, DLD‐1 were cultured in RPMI (11875093, Gibco); HL‐60, HAP1 in IMEM (A1048901, ThermoFisher); HCT116 in McCoy′s 5a (16600082, ThermoFisher) all with 10% fetal bovine serum (FBS, 10270‐106, Gibco) at 37 °C in 5% CO_2_ and 95% relative humidity.

ESCs (Human ESC Cell Line 121 (SA121), Y00025, Cellartis) were maintained on Matrigel‐coated plates (Corning) in mTeSR 1 medium (05876, STEMCELL technologies) at 37 °C, 5% CO_2_ as previously described.^[^ ^27^ ^]^ Fresh mTeSR 1 medium was replaced daily. Once the cells reached 80%‐90% confluence they were dissociated into single cells with Tryple E (12604013, ThermoFisher Scientific) at 37 °C for 5 min and seeded at a cell density of 50000 cells cm^−2^ onto new Matrigel‐coated plates in mTeSR 1 supplemented with 5 µM ROCK inhibitor Y‐27632 (1254, TOCRIS) for the first 24 h. The cell line was obtained from Takara Bio and used within passages 15–35.

Before EV isolation cells were supplemented with media with FBS that was EV‐depleted by ultracentrifugation at 100 000 × *g* (Type 45 Ti, Beckman Coulter, Brea, CA) for 16 h at 4 °C. For Expi293F cells no supplementation was necessary. EV isolations were performed with the cells viability above 80%.

All cells were mycoplasma negative and authenticated by short tandem repeat DNA profiling analysis.

### EV Isolations—Small Scale (up to 300 ml Culture Media)

Suspension cells were first pelleted by centrifugation at 300 × *g* for 10 min. Adherent cell culture supernatant was centrifuged at 300 × *g* for 10 min as well to remove detached cells. Cell debris and large EVs were removed by centrifugation at 2500 × *g* for 30 min. The cell supernatant was transferred to 94 ml quick‐seal polyallomer tubes (Beckman Coulter) and centrifuged at 20 000 x *g* for 25 min at 4 °C to pellet large vesicles followed by transfer of the supernatant to new tubes and second centrifugation at 100 000 x *g* for 2 h at 4 °C (Type 45 Ti, k‐factor 210.4, Beckman Coulter) to pellet small EVs. Culture volume was set as 300 ml for all the EVs in the biodistribution study, but hTert MSCs which were isolated from 600 ml due to expected very low yield, and ESC EVs cell culture volume was not indicated.

### EV Isolations—Large Scale (over 300 ml Culture Media)

Suspension cells were pelleted from 6 liters of culture medium by two rounds of centrifugation at 300 × *g* for 10 min. Detached adherent cells were pelleted by one round of centrifugation at 300 × *g* for 10 min. After the media was filtered with 0.22 µm filters (Corning).

To process the clarified conditioned media, an ÄKTA flux S tangential flow filtration system (GE Healthcare) was used and the protocol was adapted from.^[^ ^28^ ^]^ The media was passed through a 500 kDa cut‐off hollow fiber membrane (UPF‐500‐C‐4A, GE healthcare). The transmembrane pressure was maintained below 0.3 psi. The flow‐through with a molecular weight of less than 500 kDa was discarded, and the clarified media was circulated until it reduced in volume by 10 times (from 3 L to 300 ml). Subsequently, the sample was subjected to diafiltration with 500 ml of PBS. The retentate, which had a volume of 250 ml was collected and the fiber membrane was washed with 50 ml of PBS, which was also collected. The retentate and wash samples were combined, and the resulting volume was ultracentrifuged for 2 h 100 000 x *g* to pellet down EVs. Pellet was resuspended in PBS, aliquoted, and frozen (−80 °C) or used freshly for hEV production.

### LNP Production

The preparation procedure and formulation parameters remained unchanged for all LNPs, while the nucleic acid cargo varied for the different studies. 16 LNPs with different barcoded ssDNA (IDT, Table S3, Supporting Information) were formulated for the biodistribution study, and LNPs with Cre mRNA (Etherna) were formulated for the functional delivery study. Lipid components were: DLin‐MC3‐DMA (chemically synthesized in house), cholesterol (C8667, Sigma‐Aldrich), DSPC (LP‐R4‐076, Corden Pharma) and DMPE‐PEG_2000_ (PM‐020CN, NOF Corporation). Prior to particle assembly, the nucleic acid was solubilized in 50 mM citrate buffer pH 3 (Q2445, Teknova), and the lipids were dissolved in ethanol and mixed with a molar ratio of 50:38.5:10:1.5 (MC3:cholesterol:DSPC:DMPE‐PEG_2000_). LNPs were prepared with a NanoAssemblr Ignite (Precision NanoSystems Inc.) by mixing the two solutions at a flow rate of 12 ml min^−1^ with a 3:1 volume ratio (nucleic acid:lipid solution) to form LNPs with a lipid/nucleic acid weight ratio of 10:1. Particles were loaded into a Slide‐A‐Lyzer G2 dialysis cassette (Thermo Scientific), dialyzed in PBS pH 7.4 overnight, and then sterile filtered using a 0.2 µm filter.

The encapsulation efficiency and nucleic acid concentration were determined with either the RiboGreen (mRNA) or OliGreen (ssDNA) Assay (Thermo Fisher Scientific), according to the manufacturer's guidelines. Particle size was determined with dynamic light scattering using a DynaPro Plate Reader III (Wyatt Technology Corporation) for ssDNA LNPs, and Zetasizer Nano ZSP (Malvern Panalytical Ltd) for mRNA LNPs. Encapsulation efficiency for all LNPs was above 90% and particle size varied from 60 to 90 nm.

### hEV Production—Small‐Scale Production (for the Biodistribution Study)

Freshly prepared LNP and EVs were mixed in a 1:2 ratio based on particle count measured by nanoparticle tracking analysis (NTA) in PBS (total volume 1.5 ml). Number of EV particles used for the reaction is indicated in Table S1 (Supporting Information). Afterward, 8.5 ml of MES buffer (10 mM MES, 145mM NaCI, 5mM KCI; 0.1% DEPC, pH 5.5, sterilized by filtration through 0.22 µm filter (Corning)) was added to the mixture, gently mixed, and incubated at room temperature for 30 min. Next, the pH was adjusted to neutral (7.4) by adding 40 ml of PBS. The resulting mix was ultracentrifuged for 2 h at 100 000 x *g*. Ultracentrifugation pelleted two types of particles: dense particles resulting from the fusion events of EVs and LNPs (hEVs), and the remaining unfused EVs in the preparation. During the ultracentrifugation step, unfused LNPs were expected to be removed, as their lower density made them to float to the top of the centrifuge tube.^[^ ^29^ ^]^ Pellets were resuspended in PBS, aliquoted, and used freshly for in vivo studies.

### hEV Production—Large‐Scale Production (for the Functional Delivery Study)

Freshly prepared LNP (2.5 x 10^11^ particles per reaction) and freshly isolated EVs (5 x 10^11^ per reaction) were mixed in a 1:2 ratio based on particle count measured by nanoparticle tracking analysis (NTA) in PBS (total volume 1.5 ml). 34 reactions were set up. Same as for small‐scale production 8.5 ml of MES buffer (10 mm MES, 145mM NaCI, 5mM KCI; 0.1% DEPC, pH 5.5) was added, mixed, and incubated at room temperature for 30 min. Next, 500 µl of NaP buffer (pH 7.0) was added to adjust the pH to neutral. Maximum 9 reactions were collected in one ultracentrifugation tube (345776, Beckman Coulter) and centrifuged at 100 000 x *g* for 1 h. Pellets were resuspended in PBS and freshly used for in vivo studies.

### Protein Extraction and Immunoblot Analysis

For protein extraction and immunoblot analysis, the EV samples and cell lysates were processed as previously described.^[^ ^30^ ^]^ An equal amount of proteins per sample (30 µg) was mixed with sample buffer and heated at 70 °C for 10 min. The proteins were then separated on 4%‐12% SDS‐PAGE Bis‐Tris gels (Life Technologies) at 180 V in MES SDS running buffer and transferred using Trans‐Blot Turbo Mini or Midi polyvinylidene fluoride transfer packs (Bio‐Rad). After blocking with Odyssey TBS Blocking Buffer (LI‐COR) for 1 h at room temperature with gentle shaking. The membranes were incubated overnight at 4 °C with primary antibodies (Table S4, Supporting Information) diluted in TBS Odyssey blocking buffer. Following this, the membranes were washed with 0.1% TBS‐Tween for 5 min three times and incubated with corresponding secondary antibodies (diluted 1:20000 in 0.1% TBS‐Tween) for 1 h at room temperature. Finally, the membranes were washed three times with TBS‐Tween and visualized on the Odyssey CLx imaging system (LI‐COR), and analyzed with the Image Studio v.4.0.

### Particle Size and Concentration Measurements by Nanoparticle Tracking Analyzer

To determine the particle size and concentration of EV samples, nanoparticle tracking analysis (NTA) was employed. The NTA measurements were carried out using a NanoSight NTA instrument (Malvern Instruments). The samples were diluted in PBS and analyzed with the following settings: 40–100 particles per frame, and an acquisition time of 90 sec for three videos. The software settings were the same for all the measurements.

### Transmission Electron Microscopy and Immunogold TEM (immuno EM) Analysis

The sample preparation for the negative staining and Cryo‐electron microscopy was carried out according to previously described methods.^[^ ^31^ ^]^ For immunogold‐EM, 8 µl of isolated EVs were treated with 2% paraformaldehyde‐0.1 M PBS for 30 min, and then placed on top of carbon‐coated nickel grids for 15 min. The grids were subsequently washed in 0.1 M PBS, blocked in 0.1 M glycine 0.3% BSA for 10 min, and incubated with primary antibodies for 1–2 h. After the primary incubation, the grids were blocked for 10 min and then incubated with secondary antibodies labelled with 6 nm gold particles for 1 h. The grids were washed and imaged using an FEI Tecnai G2 Spirit (Thermo Fisher Scientific) and a digital camera Morada (Olympus Soft Image Solutions). A standard negative staining procedure using the secondary gold label antibody was performed to assess unspecific binding.

### DNA and mRNA Measurements

DNA content of hEVs was measured with Qubit single‐stranded DNA detection kit (Q33212, Life Technologies) according to the manufacturer's instructions. To measure mRNA amount Qubit RNA High Sensitivity kit (Q32852, Life Technologies) was used. To measure total DNA/mRNA content particles were lysed with 1% Triton (Sigma‐Aldrich) for 10 min. To access encapsulation efficiency, the amount of cargo associated with hybrid EVs was also measured without the addition of the detergent.

### EV Labeling

18:1 PE‐TopFluor AF488 (Avanti Research, USA) was pipetted into a glass vial and evaporated to dryness. EVs were then added to the vial at a ratio of 6 x 10^11^ particles per 1 µL of dye; the mixture was vortexed for 30 seconds and incubated at 37 °C for 30 min in the dark to allow for labeling. To remove the excess unbound dye, the labeled EV suspension was diluted to 4 mL with PBS and diafiltrated using Amicon Ultra‐4 centrifugal filter units (4 mL, 30 kDa MWCO), by centrifuging at 4100 x *g* at 4 °C, according to the manufacturer´s specifications. This step was repeated a total of 3 times to ensure removal of unbound dye. An equal reaction was prepared without EVs to validate dye removal. Successful labeling was confirmed by analyzing the percentage of AF488 positive particles using the NanoAnalyzer.

### Flow NanoAnalyzer Particle Characterization

The NanoAnalyzer (NanoFCM Co., Ltd., China) was utilized for the characterization and quantification of nanoparticles. The instrument is equipped with 488 nm and 647 nm lasers for fluorescence excitation and bandpass filters 525/40 and 670/30 for fluorescence detection were installed. Prior to sample analysis, the NanoAnalyzer was calibrated using two standard calibration beads (NanoFCM Co., Ltd.) for fluorescence intensity, concentration, and size calibration. Samples were prepared by diluting nanoparticles in filtered Tris‐EDTA buffer (TE, blank) to achieve an optimal concentration for measurement (10^8^ particles mL^−1^). Samples were measured for 120 seconds at a constant pressure of 1 kPa. For fluorescence detection, samples were illuminated with 488 nm and 647 nm lasers, both operating at a power of 10 mW. Unstained EVs and LNPs were used as controls to establish background fluorescence levels. Additionally, an empty EV staining reaction (dye only) was performed as a control for the fluorescent reagent and measured under the same conditions. Thresholds for each sample were automatically set to the average background measurements plus two times their standard deviation. The NanoViewer software (version 3.2) was employed to capture and analyze both scattering and fluorescence signals. Fluorescence data were further analyzed to identify and quantify specific subpopulations of nanoparticles labeled with fluorescent markers. To calculate the percentage of free dye, the fluorescent triggers were used, allowing the detection of fluorescent signals in the “AF488” or “Cy5” channel that were not associated with particle signals in the SS channel.

### Animals

Mouse experiments were approved by the AstraZeneca internal committee for animal studies and the Gothenburg Ethics Committee for Experimental Animals (license number 162–2015+) compliant with EU directives on the protection of animals used for scientific purposes. Male C57BL/6N (Charles River, Sulzfeld, Germany) 32 weeks old mice were individually housed in a temperature‐controlled room (21 °C) with a 12:12 h light/dark cycle and controlled humidity (45–55%). Mice had access to a regular chow diet (R36, Lactamin AB, Stockholm, Sweden) and water ad libitum. Mice were checked daily and weighed weekly. 

### Generation of Luciferase Reporter Transgenic Mice

We used a CAGGS promoter‐loxP‐Stop cassette‐loxP‐Luciferase DNA vector for the generation of the floxed luciferase reporter mice. The expression cassette was digested to remove extraneous vector sequence by agarose gel electrophoresis, purified, and injected into fertilized FVB/N mouse oocytes followed by implantation into pseudo‐pregnant females. Founder mice were screened for the presence of the transgene by PCR and subsequently bred to C57Bl6/N mice to maintain the line. Confirmation of the transgenes chromosomal position and integrity was verified by Target Locus Amplification (Cergentis).

### In Vivo Administration of hEVs—Biodistribution Study

The mix of hEVs loaded with DNA barcodes was administered intravenously to C57BL/6N mice at the dose of 0.044 mg kg^−1^. 4 h after injection, mice were sacrificed and liver, kidney, heart, lung, spleen, pancreas, brain, stomach, and intestine were collected for further analysis.

### Functional Delivery Study

Cre mRNA‐loaded hEVs were administered intravenously to LoxP‐Luc mice at the dose of 0.135 mg kg^−1^. PBS treated group was used as a control. 4 days later, D‐luciferin was administered subcutaneously, and 20 min later animals were imaged via IVIS system. Animals were then terminated, and liver, kidney, heart, lung, spleen, pancreas, brain, stomach, and intestine were collected for further analysis with luciferase assay and histology.

### Oligonucleotide Isolation and Amplification

Collected tissues (Liver, Kidney, Heart, Lung, Stomach Spleen, Pancreas, Intestine, Brain) were weighed and randomly sectioned on dry ice. 40–230 mg of tissue sample (Table S5, Supporting Information) was homogenized in 2 ml tubes containing 1.4 mm (Liver, Kidney, Lung, Spleen, Pancreas, Brain) or 2.8 mm (Heart, Intestine, Stomach) ceramic beads (Precellys) and lysis buffer (Clarity OTX, Phenomenex) by agitation for 2 x 30 s at 6000 rpm in Precellys 24 tissue homogenizer (Bertin Technologies). The tissue lysate was processed by Clarity OTX oligonucleotide isolation kit according to the manufacturer's instructions (KS0‐8494, Phenomenex) and the resulting eluate was further purified using Oligo Clean & Concentrator kit (D4061, Zymo Research). The oligonucleotides were amplified using PCR with the following parameters: 2.5 µl of oligonucleotide input, 1 µl of 10 µM forward indexing primer, 1 µl of 10 µM reverse indexing primer, 12.5 µl KAPA HiFi HotStart ReadyMix PCR Kit (Roche) in a 25 µl reaction. Indexing primers are described in Table S6 (Supporting Information). PCR amplification involved 28–33 cycles of the following settings: 98 °C for 20 seconds, 60 °C for 30 seconds, 72 °C for 30 seconds. Oligonucleotide was separated from primer dimers by isolation from 2.5% agarose gel using Nucleospin gel extraction and a clean‐up kit (Macherey‐Nagel). Separation from primer dimers and concentration of recovered samples was confirmed using Fragment Analyzer (Advanced Analytical) and Sanger sequencing prior to NGS.

### NGS

Samples with high concentrations were pooled equimolarly to 20 nM. Samples with low concentrations were pooled equimolarly to 5 nM. Three samples with very low concentrations were excluded from the pools. The concentration of both pools and the excluded samples was confirmed by measurement with high sensitivity dsDNA Qubit assay (Thermo Fisher Scientific). The pools were then further diluted and combined prior to spiking in the three samples with very low concentrations. The final pool was denatured according to guidelines provided by Illumina. The final loading concentration used as input to the sequencing was 8 pM. The samples were sequenced on a MiSeq instrument (Illumina) with a single‐end 30 bp read length setting. A total of 24.77 million pass filter reads were generated.

### In Vivo Bioluminescent Imaging

The enzymatic activity of firefly luciferase was measured by detecting the bioluminescence signal using the IVIS SpectrumCT In Vivo Imaging System (PerkinElmer). Mice were anesthetized with 3% isoflurane in an oxygen‐rich gas mixture, and injected with the RediJect D‐Luciferin Bioluminescent Substrate solution (770504, Perkin Elmer) at 150 mg kg^−1^. Bioluminescent images of dissected organs were acquired between 20 and 30 min after substrate injection. The following parameters were used for acquisition: exposure of 120 seconds, binning of 8. Bioluminescent signal was measured and represented as photons per second (P/s).

### Luciferase Measurement in Tissue Lysate

After IVIS imaging tissue samples were weighed, snap frozen in cryotubes, and kept at −80 °C until analysis. 80–120 mg of frozen tissue were homogenized in 2 ml tubes (Precellys) containing 2 mm ceramic beads in 50 µl per 10 mg of tissue of Nano‐Glo Luciferase Assay Buffer (Nano‐Glo Luciferase Assay System, Promega) by agitation for 2 x 20 s at 6000 rpm in Precellys 24 tissue homogenizer (Bertin Technologies) followed by agitation for 10 min at 25 sec^−1^ at TissueLyser ll (Qiagen). Homogenates were centrifuged at 3400 x *g* at 4 °C for 10 min and the supernatant was collected. 100 µl of the supernatant was transferred to an OptiPlate‐96 (White Opaque 96‐well Microplate, PerkinElmer, MA, USA) and 100 µl Nano‐Glo Luciferase Assay Reagent (E1500, Promega) was added. Luminescence was detected using a PHERAstar FSX microplate reader (BMG Labtech) for 5 s light collection time.

### Histology

Tissue samples were fixed in 10% NBF for 48 hr and subsequently embedded into paraffin (Milestone MAGNUS tissue processor, 4–5 mm program). Slides were baked at 60 °C for 1h. Deparaffinization, antigen retrieval (CC1 40 min, 95 °C) and immunofluorescence staining were performed on Ventana Discovery Ultra research platform (Roche). Luciferase antibody (NB100‐1677, Biotechne) signal was developed by using a goat linker (305‐005‐045, Jackson Immunology), OmniMap anti‐Rb HRP (760‐4311, Roche) and the DISCOVERY FAM Kit (Roche). The antibodies were stripped by using the standard Ventana protocol and subsequently, F4/80 antibody (70076, Cell Signaling Technology) signal was developed with OmniMap anti‐Rb HRP and the DISCOVERY Cy5 Kit (Roche). Stained slides were washed in water supplemented with dishwashing liquid to remove the oil cover slip from the Ventana system and mounted with VECTASHIELD Vibrance Antifade Mounting Medium (H‐1700, Vector Laboratories). Mounted slides were scanned with the PhenoImager HT (Akoya Biosciences) and subsequent fluorescence un‐mixing was performed in the InForm software (Akoya Biosciences). Un‐mixed images were stitched to a whole slide scan in Visiopharm and an AI tissue classification (based on DAPI and autofluorescence) was used to distinguish blood vessels, epithelium and parenchyma (Visiopharm A/S).

### NGS Data Analysis

NGS fastq files consisting of 31 bp reads were processed using simple shell and python scripts to count the number of distinct UMIs per barcode and produce a table of counts and percentages of barcodes per sample. The average number of barcodes with distinct UMIs per tissue from 4 animals was calculated for further analysis.

Next, to get individual barcode biodistribution, we normalized the number of reads corresponding to a barcode with distinct UMI found in the tissue to the size of the processed tissue. Then the sum of the normalized reads for the same barcode was set as 100%.

To compare the accumulation of the different barcodes within the same organ, we first normalized the number of reads corresponding to barcodes with unique UMIs found in the tissue to the number of barcode reads with unique UMIs in the input library. Then the sum of the normalized reads of all barcodes in one organ was set as 100%.

### Statistical Analysis

The type of presented data and number of replicates are indicated in the figure legends. Statistical analyses were done in R software using one‐way ANOVA test.

For individual barcode biodistribution across different tissues (Figure 3b) we first normalized the number of all reads with unique UMIs corresponding to a barcode found in the tissue to the size of the processed tissue. After we calculated the sum of the normalized reads for each specific barcode for the same organ for all the animals. Next, we set the sum of the normalized reads for the same barcode to 100%.

To calculate the distribution of different barcodes within the same organ, we first normalized the number of all reads with unique UMIs corresponding to barcodes found in the tissue to the number of the barcode reads with unique UMIs in the hEVs library before injection. Then the sum of the normalized reads of all barcodes in a given organ was set as 100%.

Biorender.com was used to generate panel A on Figure 3.

## Conflict of Interest

All authors are current or former employees of AstraZeneca.

## Author Contributions

A.I. and N.D. conceived and planned the experiments. A.I., J.W., and N.D. designed in vivo work. A.I., R.C., A.F.L., H.G.‐K.G., I.V., and X.W. performed the experiments. E.O.B., E.L.I., and A.T. facilitated and prepared LNPs. J.L. supported NGS sample preparation and sequencing. M.F. processed NGS data. R.C., H.G.‐K.G., L.H., F.K., and G.M. performed in vivo work. L.S. supported histology work. A.F.L. performed experiments with NanoAnalyzer. A.S. and K.J. provided scientific input to the project. A.I. analyzed and summarized the data and prepared the figures. A.I. and N.D. prepared the manuscript with input from all coauthors. All the authors contributed to the scientific discussions.

## Supporting information



Supporting Information

## Data Availability

Additional information can be found in supplementary material and be made available upon request.
