# Supplementary material for: Barcoded Hybrids of Extracellular Vesicles and Lipid Nanoparticles for Multiplexed Analysis of Tissue Distribution
Source: Adv Sci (Weinh). 2025 Jan 16;12(10):2407850. doi: 10.1002/advs.202407850 (PMC11904941; doi:10.1002/advs.202407850)
Supplement: Supplementary file 1 — Supporting Information [file ADVS-12-2407850-s001.docx]

**Barcoded hybrids of extracellular vesicles and lipid nanoparticles for multiplexed analysis of tissue distribution**

*Alena Ivanova*, Renata Chalupska, Ana Filipa Louro, Mike Firth, Hernán González-King Garibotti, Leif Hultin, Franziska Kohl, Elisa Lázaro-Ibáñez, Julia Lindgren, Gentian Musa, Erik Oude Blenke, Andreia Silva, Louis Szeponik, Agnes Taylor, Ida Viken, Xiaoqin Wang, Karin Jennbacken, John Wiseman, Niek Dekker**

**Supplementary materials**

**
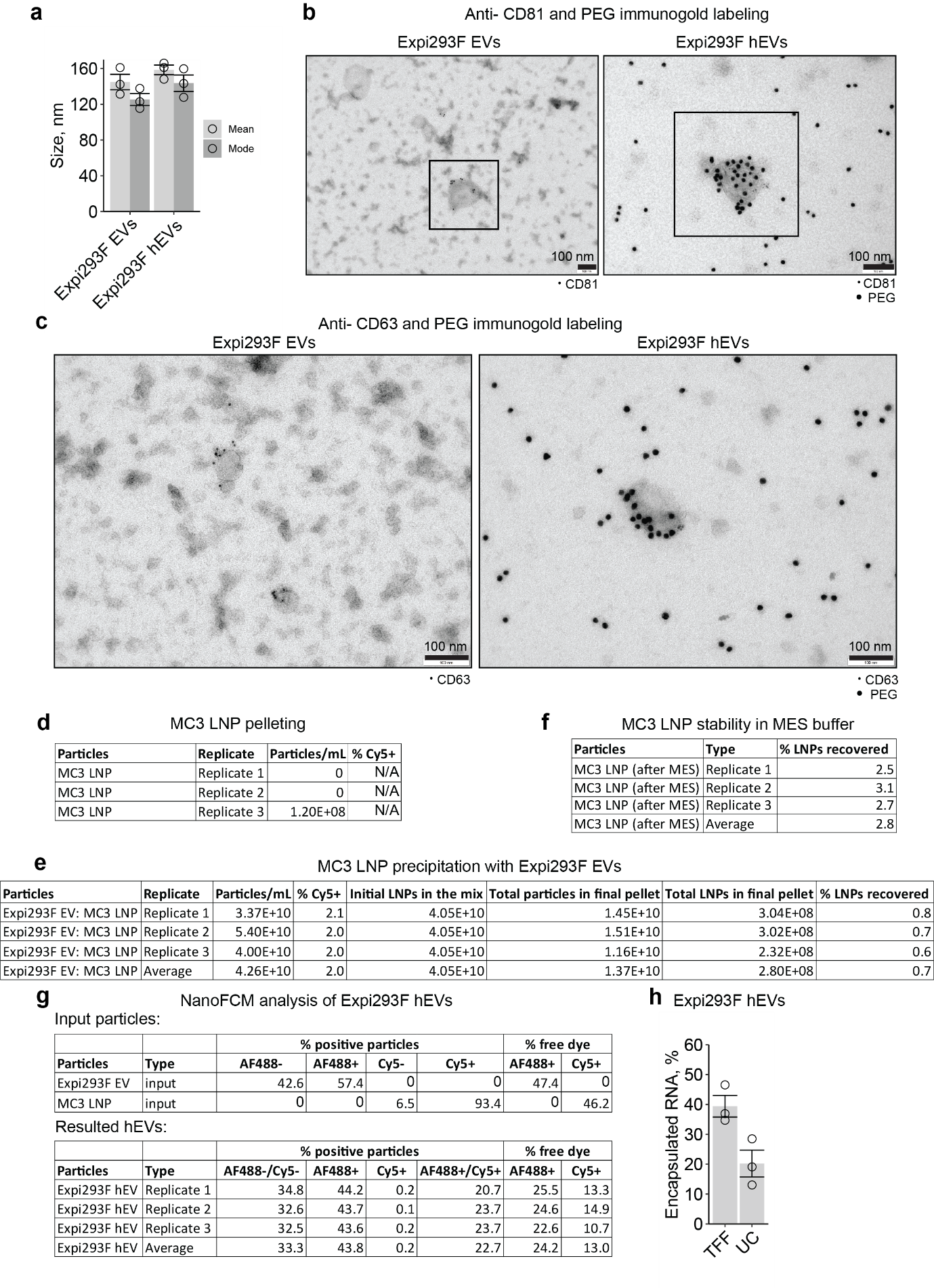
**

**Supplementary Figure S1. Characterization of hEVs originated from Expi293F cells.**

**a)** Mean and mode sizes of EVs isolated from Expi293F cells and hEVs produced from them. Calculated based on nanoparticle tracking analysis. An average of 3 measurements ± s.e.m. is shown.

**b)** Representative immuno-gold negative staining transmission electron microscopy (TEM) wide-field images of EVs isolated from Expi293F cells and hEVs produced from them. EVs were incubated with primary and secondary antibodies conjugated with 6 nm gold particles (for CD81). hEVs were additionally incubated with primary and secondary antibodies conjugated with 15 nm gold particles (for PEG). Representative EV images depict CD81-positive EVs and PEG^+^CD81^+^ double-positive hEVs. Scale bar is = 100 nm. Zoomed-in sections shown in Figure 1F are denoted with a black box.

**c)** Immunogold labeling of EVs isolated from Expi293F cells and hEVs produced from them. EVs were incubated with primary and secondary antibodies conjugated with 6 nm gold particles (for CD63). hEVs were additionally incubated with primary and secondary antibodies conjugated with 15 nm gold particles (for PEG). Representative EV images depict CD63-positive EVs and CD63^+^PEG^+^ double-positive hEVs. Scale bar is = 100 nm.

**d), e)** MC3 LNPs carrying Cy5-labeled mRNA were pelleted via ultracentrifugation at 100,000 x g for 2 hours alone **(d)** or together with Expi293F EVs **(e)**. The concentration of recovered particles and Cy5+ particles was measured with Flow NanoAnalyzer. The particle concentration of LNPs pelleted without EVs was at the detection limit of Flow NanoAnalyzer, and Cy5+ particles were not found (indicated as N/A). Data shown represent three replicates of the experiment.

**f)** MC3 LNPs carrying Cy5-labeled mRNA were incubated in TE or in MES buffer for 30 min. After incubation, particle concentration and the percentage of Cy5+ particles were measured with Flow NanoAnalyzer. The decrease in particle concentration in MES buffer was calculated relative to the TE buffer. Data shown represent three replicates of the experiment.

**g)** Flow NanoAnalyzer analysis of Expi293F hEVs. Expi293F EVs were labeled with PE-TopFluoro AF488 dye. MC3 LNPs carried Cy5-labeled mRNA. hEV particles were formulated as discussed previously. Additionally, the percentage of unbound dye in the EV sample and free Cy5-labeled mRNA is indicated. Data shown represent three replicates of the experiment.

**h)** Comparison of mRNA encapsulation efficiency in hEVs produced using EVs purified with two different techniques: tangential flow filtration (TFF) and ultracentrifugation (UC).

**
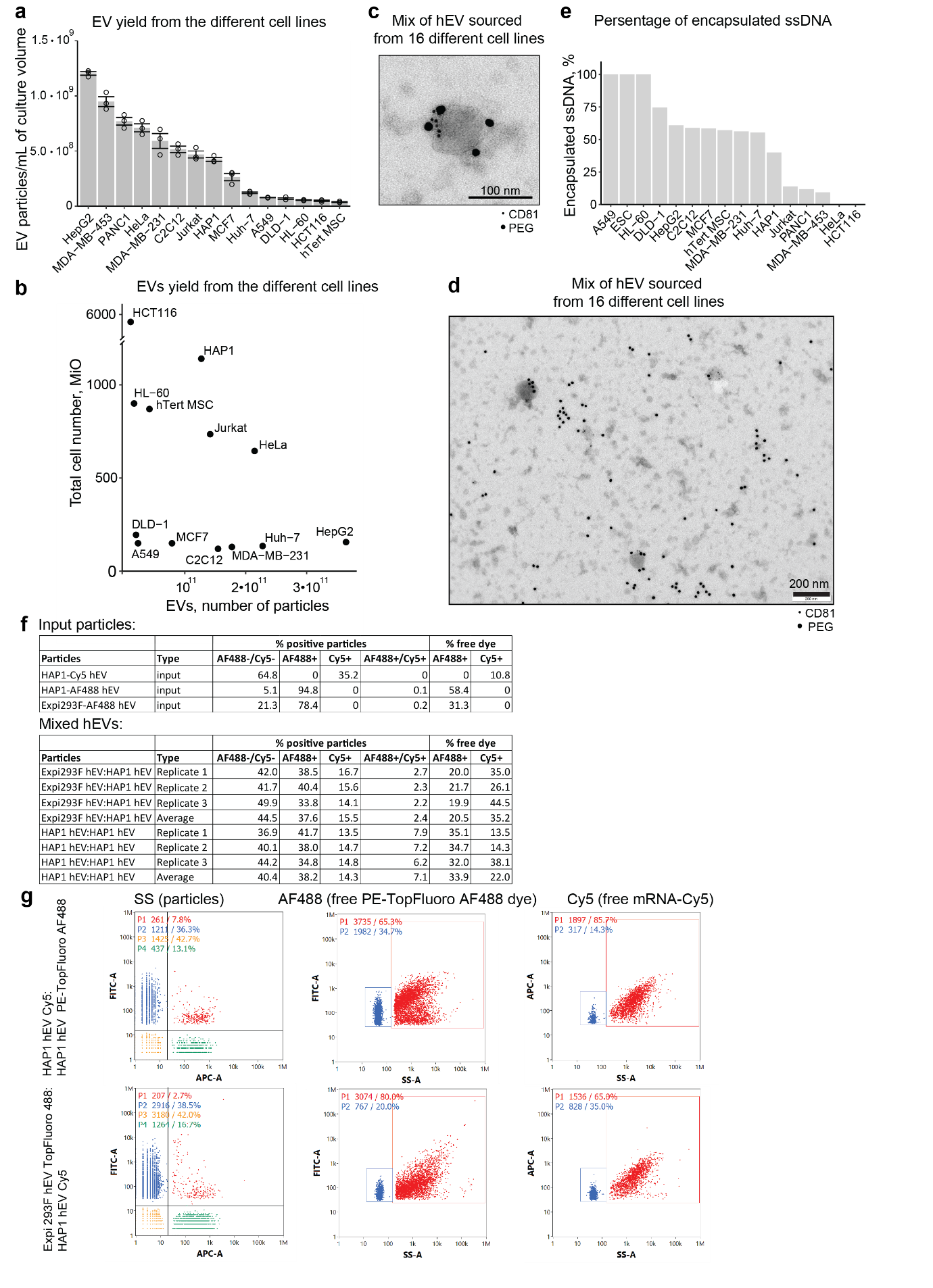
**

**Figure S2. Characterization of EVs and hEVs for multiplexed DNA barcoding experiment**

**a)** Indicated is EV yield from different cell lines. The average of three measurements of the number of particles ± s.e.m. per ml of culture.

**b)** EV yield from the different cell lines is indicated. The cell culture medium was changed to the media with EV-depleted serum 24 hours prior to EV isolation. The number of cells at the time of EV collection is plotted on the y-axis, and the number of collected EVs is plotted on the x-axis.

**c)** Immunogold labeling of a mix of 16 hEVs produced from 16 different cell lines. hEVs were incubated with primary and secondary antibodies conjugated with 6 nm (for CD81) and with 15 nm (for PEG) gold particles. The representative image depicts a double-positive for CD81 and PEG hEV. Scale bar is = 100 nm.

**d)** Representative immuno-gold negative staining transmission electron microscopy (TEM) wide-field images of hEVs produced from 16 different EVs. hEVs were incubated with primary and secondary antibodies conjugated with 6 nm (for CD81) and with 15 nm (for PEG) gold particles. The representative image depicts a double-positive for CD81 and PEG hEV. Scale bar is = 100 nm.

**e)** Characterization of designed hEVs. The encapsulated amount of DNA barcodes was calculated based on the measurement of total ssDNA in the presence or absence of the detergent (N=1).

**f), g)** Flow NanoAnalyzer analysis of the indicated mixes of hEV particles. The first mix (HAP1:HAP1) contained HAP1 hEVs labeled with PE-TopFluoro AF488 dye together with HAP1 hEVs containing Cy5-labeled mRNA. The second mix (Expi293F:HAP1) contained Expi293F hEVs labeled with PE-TopFluoro AF488 membrane dye and HAP1 hEVs containing Cy5-labeled mRNA. Additionally, the percentage of unbound dye in the EV sample and free Cy5-labeled mRNA is indicated. Data shown represent three replicates of the experiment.

**
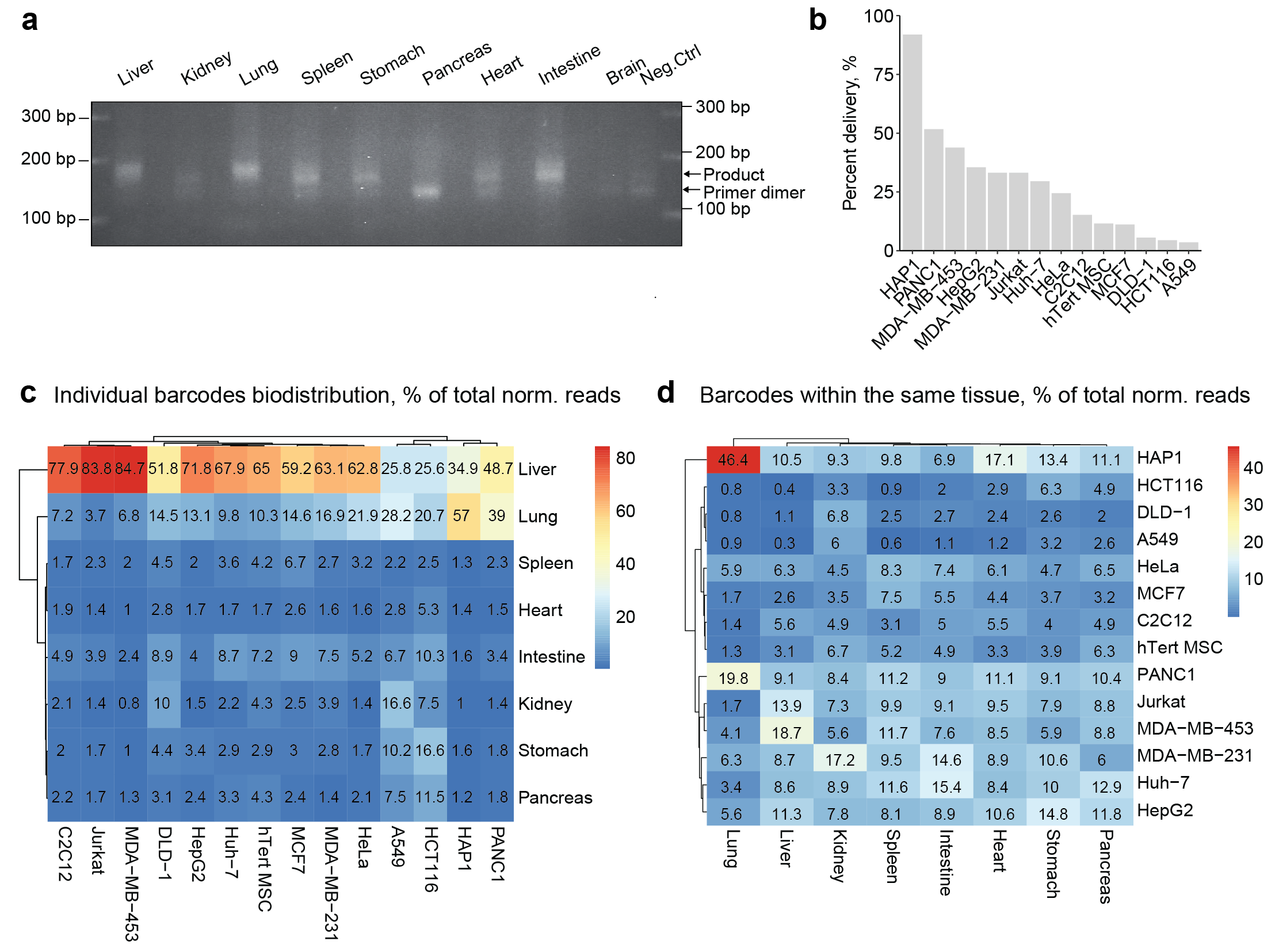
**

**Figure S3. Barcode amplification and delivery to different tissues.**

**a)** Representative agarose gel for amplified and indexed barcodes. Upper bands correspond to the size of the indexed barcode. Lower bands (below the dashed line) are primer dimers. Water was used as an input for the PCR reaction for the negative control (Neg. Ctrl) condition. Expected product size is 156 bp.

**b)** Percentage of injected unique DNA counts found in mice 4 hours post-injection. To calculate the hEV delivery percentage, the number of barcode counts detected in the tissue is divided by the total number of unique DNA counts for each barcode in the library before injection. The total number of unique DNA counts for each barcode was determined by sequencing the input library.

**c)** Individual barcode biodistribution across examined tissues. The total number of normalized unique counts for each barcode was set as 100%.

**d)** Barcode representation within defined tissues. All normalized unique counts identified within the tissue were set as 100%.

**
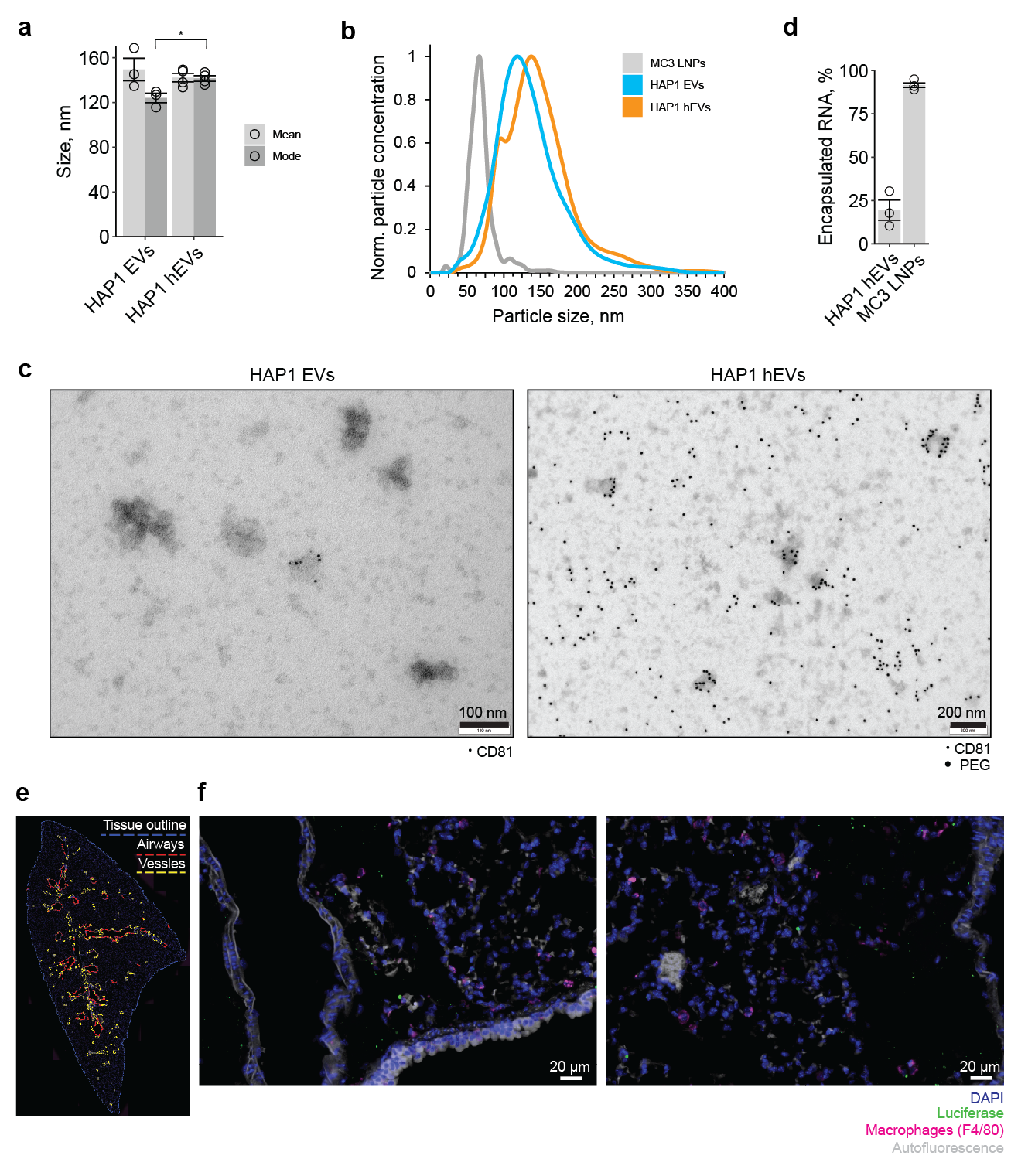
**

**Supplementary Figure S4. Characterization of HAP1-originated hEVs.**

**a)** Mean and mode sizes of EVs isolated from HAP1 cells and hEVs produced from them. Calculated based on nanoparticle tracking analysis. Shown is an average of 3 (for EVs) or 4 (for hEVs) independent experiments ± s.e.m). The P-value was calculated using one-way ANOVA statistical test. The asterix indicates P<0.05.

**b)** Nanoparticle tracking analyses of LNPs (grey line), HAP1 EVs (dark orange line), and hEVs (light orange line). Representative size distribution and particle concentration graphs are shown.

**c)** Representative immuno-gold negative staining transmission electron microscopy (TEM) wide-field images of EVs isolated from Expi293F cells and hEVs produced from them. EVs were incubated with primary and secondary antibodies conjugated with 6 nm (for CD81). hEVs were additionally incubated with primary and secondary antibodies conjugated with 15 nm (for PEG) gold particles. Scale bar is = 100 nm.

**d)** Amount of encapsulated mRNA in the HAP1 hEVs and MC3 LNPs. The total amount of mRNA associated with hEVs or LNPs was set as 100%. An average of three (for HAP1 hEVs) and 4 (for LNPs) measurements ± s.e.m. is shown.

**e) – f)** Representative immunohistology images of lung tissue from the animals treated with PBS.

**e)** Lung tissue section. Dotted lines indicate tissue outline (blue), airways (red), and blood vessels (yellow).

**f)** Lung tissue was stained with the DAPI nuclear dye (blue), luciferase antibodies (green), F4/80 antibodies to detect macrophages (magenta), and tissue autofluorescence was detected to identify blood vessels (grey). Scale bar is = 20 μm.

**Table S1.** Particle number in the fusion reaction.

| EV | Number of EVs in the fusion reaction |
| --- | --- |
| MDA-MB-231 | 1.77·10^11^ |
| Huh-7 | 2.28·10^11^ |
| HegG2 | 3.65·10^11^ |
| HeLa | 2.15·10^11^ |
| MCF7 | 7.89·10^10^ |
| DLD-1 | 2.00·10^10^ |
| A549 | 2.33·10^10^ |
| C2C12 | 1.55·10^11^ |
| HCT116 | 1.48·10^10^ |
| HAP1 | 1.27·10^11^ |
| HL-60 | 1.67·10^10^ |
| Jurkat | 1.42·10^11^ |
| hTert MSC | 4.20·10^10^ |
| PANC1 | 2.34·10^11^ |
| MDA-MB-453 | 2.85·10^11^ |
| ESC | 1.80·10^10^ |

**Table S2.** Injected doses based on the total ssDNA in the particle mix.

| N | Sample name | Dose, % from injected total ssDNA | Dose, mg/kg of total ssDNA |
| --- | --- | --- | --- |
| 1 | MDA-MB-231 | 28.82 | 1.28 · 10^-2^ |
| 2 | Huh-7 | 3.52 | 1.6 · 10^-3^ |
| 3 | HepG2 | 3.77 | 1.7 · 10^-3^ |
| 4 | HeLa | 8.36 | 3. · 10^-3^ |
| 5 | MCF7 | 0.72 | 3 · 10^-4^ |
| 6 | DLD-1 | 0.2 | 9 · 10^-5^ |
| 7 | A549 | 0.06 | 3 · 10^-5^ |
| 8 | C2C12 | 21.42 | 9.5 · 10^-3^ |
| 9 | HCT116 | 0.07 | 3 · 10^-5^ |
| 10 | HAP1 | 2.72 | 1.2 · 10^-3^ |
| 11 | HL-60 | 0.03 | 1 · 10^-5^ |
| 12 | Jurkat | 9.67 | 4.3 · 10^-3^ |
| 13 | hTert MSC | 0.05 | 2 · 10^-5^ |
| 14 | PANC1 | 14.1 | 6.3 · 10^-3^ |
| 15 | MDA-MB-453 | 6.48 | 2.9 · 10^-3^ |
| 16 | ESC | 0.02 | 1 · 10^-5^ |

**Table S3.** Barcodes sequences.

| Barcode N | Sample name | Barcode sequence |
| --- | --- | --- |
| Barcode 1 | MDA-MB-231 | A*G*A*CGTGTGCTCTTCCGATCT GAGGGTACTT NNNNNNNNNN AGATCGGAAGAGCGTCG*T*G*T |
| Barcode 2 | Huh-7 | A*G*A*CGTGTGCTCTTCCGATCT GACAATTGCC NNNNNNNNNN AGATCGGAAGAGCGTCG*T*G*T |
| Barcode 3 | HepG2 | A*G*A*CGTGTGCTCTTCCGATCT TAACGCACCT NNNNNNNNNN AGATCGGAAGAGCGTCG*T*G*T |
| Barcode 4 | HeLa | A*G*A*CGTGTGCTCTTCCGATCT ATGATCGTCG NNNNNNNNNN AGATCGGAAGAGCGTCG*T*G*T |
| Barcode 5 | MCF7 | A*G*A*CGTGTGCTCTTCCGATCT TGTCTCCCAT NNNNNNNNNN AGATCGGAAGAGCGTCG*T*G*T |
| Barcode 6 | DLD-1 | A*G*A*CGTGTGCTCTTCCGATCT GGAGAAACAG NNNNNNNNNN AGATCGGAAGAGCGTCG*T*G*T |
| Barcode 7 | A549 | A*G*A*CGTGTGCTCTTCCGATCT CGTACAAACG NNNNNNNNNN AGATCGGAAGAGCGTCG*T*G*T |
| Barcode 8 | C2C12 | A*G*A*CGTGTGCTCTTCCGATCT GATTTGTGGG NNNNNNNNNN AGATCGGAAGAGCGTCG*T*G*T |
| Barcode 9 | HCT116 | A*G*A*CGTGTGCTCTTCCGATCT TTGCAGCCTT NNNNNNNNNN AGATCGGAAGAGCGTCG*T*G*T |
| Barcode 10 | HAP1 | A*G*A*CGTGTGCTCTTCCGATCT GAATGCTGAC NNNNNNNNNN AGATCGGAAGAGCGTCG*T*G*T |
| Barcode 11 | HL-60 | A*G*A*CGTGTGCTCTTCCGATCT ATCCATGAGG NNNNNNNNNN AGATCGGAAGAGCGTCG*T*G*T |
| Barcode 12 | Jurkat | A*G*A*CGTGTGCTCTTCCGATCT TTCCACGATG NNNNNNNNNN AGATCGGAAGAGCGTCG*T*G*T |
| Barcode 13 | hTert MSC | A*G*A*CGTGTGCTCTTCCGATCT GCTGGGAATT NNNNNNNNNN AGATCGGAAGAGCGTCG*T*G*T |
| Barcode 14 | PANC1 | A*G*A*CGTGTGCTCTTCCGATCT CAAAACGACG NNNNNNNNNN AGATCGGAAGAGCGTCG*T*G*T |
| Barcode 15 | MDA-MB-453 | A*G*A*CGTGTGCTCTTCCGATCT TCTCGCCTTT NNNNNNNNNN AGATCGGAAGAGCGTCG*T*G*T |
| Barcode 16 | ESC | A*G*A*CGTGTGCTCTTCCGATCT CAGATCAGAG NNNNNNNNNN AGATCGGAAGAGCGTCG*T*G*T |

The * refers to a phosphorothioate-modified linkage, and N’s represent random nucleotides.

**Table S4.** Antibodies for Western blotting.

| N | Antibody | Source | Identifier | Comments |
| --- | --- | --- | --- | --- |
| 1 | Alix | Abcam | ab117600 | Application WB, dilution 1:1000 |
| 2 | TSG101 | Abcam | ab30871 | Application WB, dilution 1:1000 |
| 3 | CD81 | Abcam | ab79559 | Immuno EM, dilution 1:50 |
| 4 | CD63 | Abcam | ab59479 | Immuno EM, dilution 1:50 |
| 5 | Calnexin | Abcam | ab22595 | Application WB, dilution 1:1000 |
| 6 | Syntenin | Abcam | ab19903 | Application WB, dilution 1:1000 |
| 7 | PEG | Abcam | ab190652 | Application WB, dilution 1:1000; Immuno EM, dilution 1:50 |
| 8 | b-Actin | Sigma-Aldrich | A1978 | Application WB, dilution 1:2000 |
| 9 | Goat anti Mouse 680 | Licor | 925-68070 | Application WB, dilution 1:20000 |
| 10 | Goat anti Mouse 800 | Licor | 926-32210 | Application WB, dilution 1:20000 |
| 11 | Goat anti Rabbit 680 | Licor | 926-68071 | Application WB, dilution 1:20000 |
| 12 | Goat anti Rabbit 800 | Licor | 926-32211 | Application WB, dilution 1:20000 |

**Table S5.** Weight of tissues used for the isolation of barcodes.

| Animal N | Tissue | Weight, mg |
| --- | --- | --- |
| #1 | Liver | 230 |
| #1 | Kidney | 140 |
| #1 | Lung | 90 |
| #1 | Spleen | 50 |
| #1 | Pancreas | 50 |
| #1 | Stomach | 90 |
| #1 | Intestine | 170 |
| #1 | Brain | 170 |
| #1 | Heart | 90 |
| #2 | Liver | 200 |
| #2 | Kidney | 150 |
| #2 | Lung | 90 |
| #2 | Spleen | 40 |
| #2 | Pancreas | 70 |
| #2 | Stomach | 110 |
| #2 | Intestine | 120 |
| #2 | Brain | 130 |
| #2 | Heart | 80 |
| #3 | Liver | 300 |
| #3 | Kidney | 150 |
| #3 | Lung | 80 |
| #3 | Spleen | 40 |
| #3 | Pancreas | 70 |
| #3 | Stomach | 90 |
| #3 | Intestine | 130 |
| #3 | Brain | 160 |
| #3 | Heart | 100 |
| #4 | Liver | 120 |
| #4 | Kidney | 150 |
| #4 | Lung | 90 |
| #4 | Spleen | 40 |
| #4 | Pancreas | 70 |
| #4 | Stomach | 90 |
| #4 | Intestine | 200 |
| #4 | Brain | 120 |
| #4 | Heart | 70 |

**Table S6.** Primers for amplification and indexing for NGS.

| Primer Name | Primer |
| --- | --- |
| D501R | AATGATACGGCGACCACCGAGATCTACACAGGCTATAACACTCTTTCCCTACACGACGCTCTTCCGATCT |
| D502_R | AATGATACGGCGACCACCGAGATCTACACGCCTCTATACACTCTTTCCCTACACGACGCTCTTCCGATCT |
| D503_R | AATGATACGGCGACCACCGAGATCTACACAGGATAGGACACTCTTTCCCTACACGACGCTCTTCCGATCT |
| D504_R | AATGATACGGCGACCACCGAGATCTACACTCAGAGCCACACTCTTTCCCTACACGACGCTCTTCCGATCT |
| D505_R | AATGATACGGCGACCACCGAGATCTACACCTTCGCCTACACTCTTTCCCTACACGACGCTCTTCCGATCT |
| D506_R | AATGATACGGCGACCACCGAGATCTACACTAAGATTAACACTCTTTCCCTACACGACGCTCTTCCGATCT |
| D507_R | AATGATACGGCGACCACCGAGATCTACACACGTCCTGACACTCTTTCCCTACACGACGCTCTTCCGATCT |
| D508_R | AATGATACGGCGACCACCGAGATCTACACGTCAGTACACACTCTTTCCCTACACGACGCTCTTCCGATCT |
| D701_F | CAAGCAGAAGACGGCATACGAGATCGAGTAATGTGACTGGAGTTCAGACGTGTGCTCTTCCGATCT |
| D702_F | CAAGCAGAAGACGGCATACGAGATTCTCCGGAGTGACTGGAGTTCAGACGTGTGCTCTTCCGATCT |
| D703_F | CAAGCAGAAGACGGCATACGAGATAATGAGCGGTGACTGGAGTTCAGACGTGTGCTCTTCCGATCT |
| D704_F | CAAGCAGAAGACGGCATACGAGATGGAATCTCGTGACTGGAGTTCAGACGTGTGCTCTTCCGATCT |
| D705_F | CAAGCAGAAGACGGCATACGAGATTTCTGAATGTGACTGGAGTTCAGACGTGTGCTCTTCCGATCT |
| D706_F | CAAGCAGAAGACGGCATACGAGATACGAATTCGTGACTGGAGTTCAGACGTGTGCTCTTCCGATCT |
| D707_F | CAAGCAGAAGACGGCATACGAGATAGCTTCAGGTGACTGGAGTTCAGACGTGTGCTCTTCCGATCT |
| D708_F | CAAGCAGAAGACGGCATACGAGATGCGCATTAGTGACTGGAGTTCAGACGTGTGCTCTTCCGATCT |
| D709_F | CAAGCAGAAGACGGCATACGAGATCATAGCCGGTGACTGGAGTTCAGACGTGTGCTCTTCCGATCT |
| D710_F | CAAGCAGAAGACGGCATACGAGATTTCGCGGAGTGACTGGAGTTCAGACGTGTGCTCTTCCGATCT |
| D711_F | CAAGCAGAAGACGGCATACGAGATGCGCGAGAGTGACTGGAGTTCAGACGTGTGCTCTTCCGATCT |
| D712_F | CAAGCAGAAGACGGCATACGAGATCTATCGCTGTGACTGGAGTTCAGACGTGTGCTCTTCCGATCT |
